# Deformation‐Induced Formation of Stray Grains in Additive Manufacturing of Single Crystals

**DOI:** 10.1002/advs.202522704

**Published:** 2026-02-15

**Authors:** Dongsheng Zhang, Zixu Guo, Yuxiao Li, Lu Wang, Yu Wu, Darui Sun, Wentao Yan, Yuanyuan Guo, Han Wang, Wei Liu, Ye Tao, Bingbing Zhang

**Affiliations:** ^1^ Institute of High Energy Physics Chinese Academy of Sciences Beijing China; ^2^ Department of Mechanical Engineering National University of Singapore Singapore Singapore; ^3^ 3D Printing Research and Engineering Technology Center Beijing Institute of Aeronautical Materials Beijing China; ^4^ Science and Technology on Advanced High Temperature Structural Materials Laboratory Beijing Institute of Aeronautical Materials Beijing China; ^5^ University of Chinese Academy of Sciences Beijing China

**Keywords:** additive manufacturing, in situ monitoring, multi‐physics simulation, single crystal, synchrotron radiation

## Abstract

The formation of stray grains (SGs) remains a critical and pervasive challenge hindering the additive manufacturing (AM) of single crystals for high‐temperature aerospace applications. Here, we elucidate the mechanism underlying SG formation during the AM of Ni‐based single‐crystal alloys, through integrating in situ synchrotron imaging/diffraction, ex situ characterization, and multi‐physics modeling. In contrast to the conventional understanding that attributes SG formation solely to thermal effects, we demonstrate that SG originates from subgrain rotation driven by heterogeneous dislocation activity. We further reveal that dislocation‐induced SG formation can be regulated by substrate orientations, in which the Gini coefficient derived from dislocation distributions is proposed to serve as the physics‐based predictive metric for SG susceptibility. Specifically, high‐symmetry orientations exhibiting low Gini coefficients suppress SGs via more uniform dislocation distribution. This study advances the understanding of SG formation under extreme nonequilibrium solidification processes, thereby guiding the fabrication of high‐quality AM single‐crystal components for aerospace applications.

## Graphical abstract

Stray grain formation severely limits the additive manufacturing of single‐crystal alloys. By integrating in situ synchrotron techniques, ex situ characterization, and multi‐scale multi‐physics modeling, the authors reveal that stray grains originate from dislocations at the solid‐liquid interface, rather than thermal supercooling in conventional understanding. A dislocation‐based Gini coefficient is proposed to predict the susceptibility of stray grain formation.

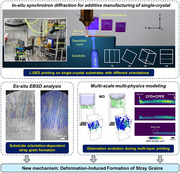

## 1 Introduction

Single‐crystal (SX) superalloys exhibit superior high‐temperature mechanical properties, which are commonly applied to hot components in aero‐engines [1]. Laser‐directed energy deposition (L‐DED), an additive manufacturing (AM) technique, offers the potential to directly fabricate SX components with intricate heat dissipation structures to enhance their operating temperatures [2, 3]. However, fabricating SX components using L‐DED poses a significant challenge in maintaining continuous and stable epitaxial growth, due to the formation of stray grains (SGs) [4], which have a detrimental effect on the resistance to creep and high‐temperature fatigue, since the high‐angle grain boundaries are prone to sliding and nucleating cracks at high temperatures [5]. Despite some efforts on the mitigation of SGs during the AM process, such as controlling substrate orientation [6], optimizing printing parameters [7, 8], or modifying printing strategy [9, 10]. Those attempts mainly rely on trial‐and‐error experiments without an in‐depth understanding of the SG formation mechanism. Under the AM process with a high cooling rate and large temperature gradient, the lack of SG formation mechanism fundamentally hinders the development of universal strategies for SX printing at extreme solidification conditions.

Conventional theories attribute the formation of SGs during the AM process to the constitutional supercooling widely recognized in the solidification community [11]. According to this theory, the upper regions of the liquid phase are prone to undercooling, leading to the formation of randomly oriented equiaxed grains [12, 13]. This only explains the formation of equiaxed SGs at the surface of AM‐produced parts, without accounting for the formation of columnar SGs deeper within the melt pool. Once thermal conditions favor undercooling, extensive homogeneous nucleation occurs at the top of the melt pool, where the growth of equiaxed grains competes with each other, interrupting the development of larger columnar grains. This also implies that the formation of columnar SGs cannot be understood from a conventional mechanism. To gain deeper insight into the mechanisms underlying the columnar SG formation, particularly to clarify the origin and evolution of columnar SGs, it is imperative to employ advanced in situ characterization techniques such as synchrotron radiation X‐ray imaging and diffraction. Synchrotron X‐ray imaging enables real‐time, high‐resolution observation of melt pool dynamics, keyhole behavior, and pore evolution [14, 15, 16, 17], while X‐ray diffraction provides quantitative data on grain structure, strain tensor evolution, and crystal defect formation during rapid solidification [18, 19, 20, 21].

In our previous work, we used an operando synchrotron Laue diffraction technique to visualize the growth behavior of the grain in Ni‐based SX under both laser remelting and single‐layer laser melting modes [22]. With a temporal resolution of 5 ms afforded by the 3W1 beamline at Beijing Synchrotron Radiation Facility (BSRF), we demonstrated that deformation essentially exists under the laser‐induced non‐equilibrium solidification process, and found some evidence on deformation‐induced SG formation in the AM process. This observation is in contrast with the formation mechanism in the conventional SX fabrication method, with a slow solidification rate, minimal thermal stress, and plastic deformation during the manufacturing process. In the field of condensed matter physics, Yankova et al. discussed the competition between surface/growth stress and epitaxial strain, emphasizing that this competition is strongly orientation‐dependent and gives rise to local microstructural variations, including defect formation and orientation deviations [23]. Extending this concept to SX additive manufacturing, we infer that the intrinsic anisotropy of the SX substrate similarly governs the deformation response. This anisotropy‐driven variation in deformation accommodation directly regulates the likelihood of SG formation, thereby providing a mechanistic link between substrate orientation, stress evolution, and deformation‐induced SG development. However, this potential mechanism is not experimentally verified, and its applicability to the multi‐layer AM process is still unexplored.

In the present work, we conducted a comprehensive investigation using the Test beamline of High Energy Photon Source (HEPS) and 3W1 beamline of BSRF, utilizing in situ X‐ray imaging and Laue diffraction to unveil the influence of different substrate orientations on grain solidification, deformation, and SG formation during the L‐DED process. Our results revealed the critical role of dislocations in SG formation during the AM process. By integrating ex situ characterization, in situ experiments, lattice‐scale simulations, and multi‐physics modeling, we systematically validated the underlying mechanism of SG formation and proposed a general law for SG suppression under extreme solidification conditions.

## 2 Results

### 2.1 Operando Experiments for Grain Growth Behavior

**FIGURE 1:**
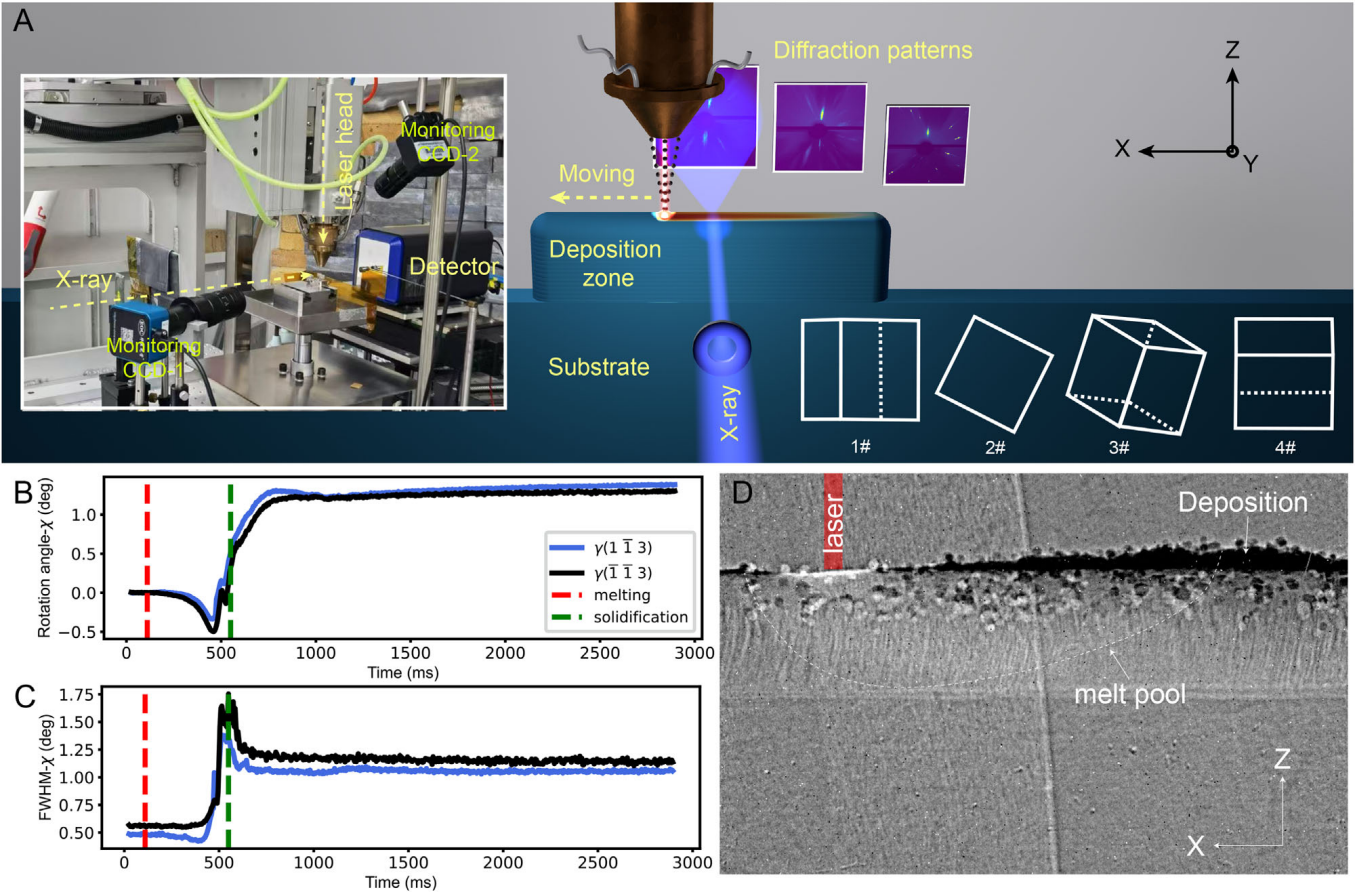
In situ detection system during L‐DED. (A) Diagram of the operando Laue diffraction system. The picture of the operando Laue diffraction system is shown in the inset. (B,C) The rotation angle (B) and FWHM (C) in the χ direction versus time during the first‐layer L‐DED for 1#‐(001)/[$1\bar{2}0$]. The blue and black dots represent the γ($1\bar{1}3$) and γ($\bar{1}\bar{1}3$) lattice planes, respectively. The red and blue dashed lines represent the start and stop times of the AM process. (D) A transient radiograph acquired during the fourth‐layer L‐DED process, which is an X‐ray image after image processing through dividing the brightness at each pixel of Frame i by the brightness of corresponding pixels in Frame 1 (initial state).

The temperature gradient and solidification velocity along the dendrite growth direction are directly influenced by the local growth orientation at the solidification front. Consequently, the substrate orientation has a profound effect on the formation of SGs in laser‐based AM of SX superalloys [24, 25]. To systematically investigate the relationship among substrate orientation, deformation, and SG formation, we conducted, for the first time, the in‐depth operando Laue diffraction and X‐ray imaging study of the L‐DED process using four representative substrate orientations (listed in Table S1) at the BSRF and HEPS. The experimental setup and methodology are described in detail in the Methods Section. The operando platform and crystallographic schematic diagrams of four substrate orientations are illustrated in Figure 1A. The four‐orientation operando Laue diffraction patterns, which varied with the number of deposited layers, are shown in Movies S1–S4.

**FIGURE 2:**
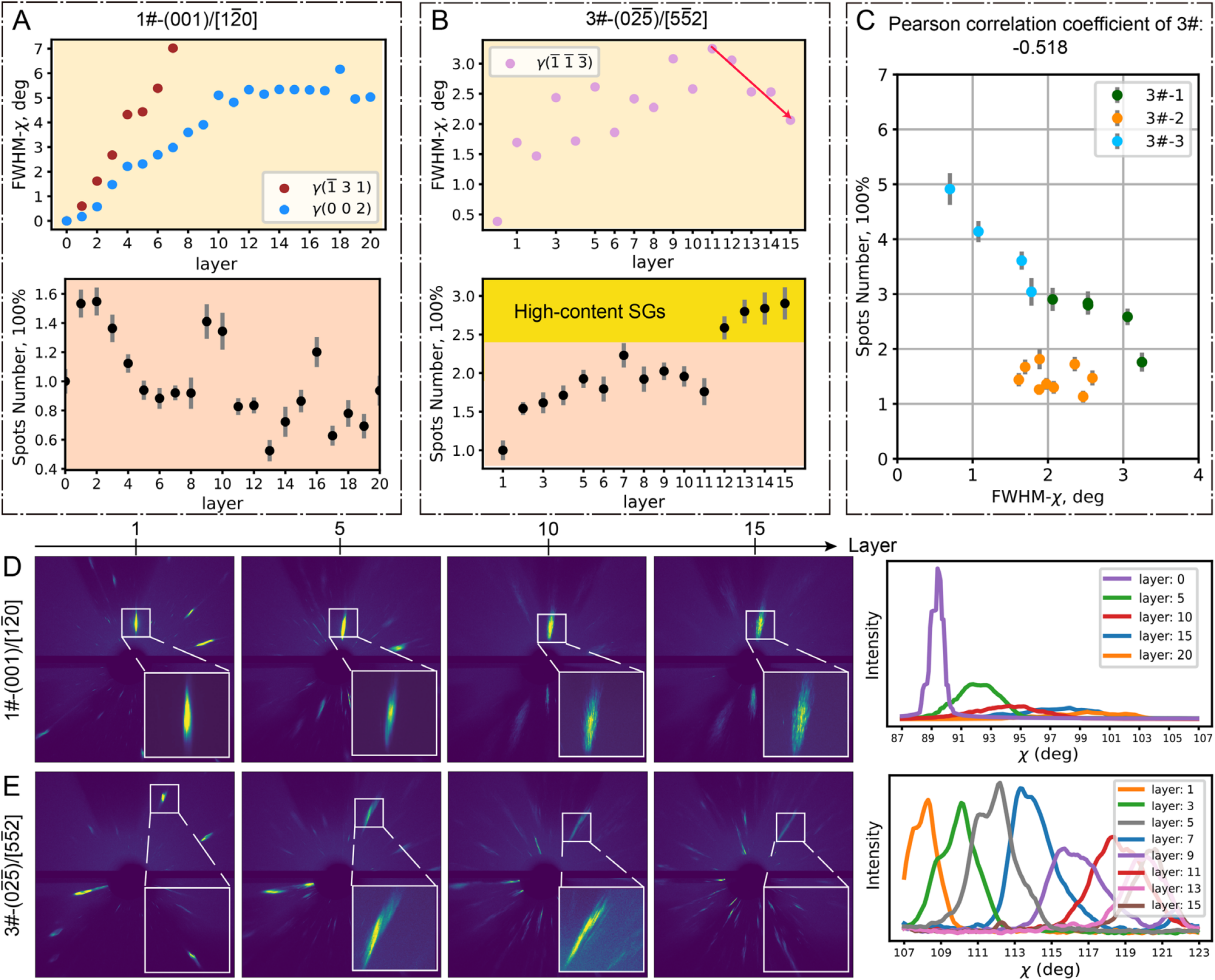
SX growth behavior on substrates with different orientations. (A,B) Evolution of FWHM and spot number as a function of increasing printing layers during the L‐DED process for 1#‐(001)/[$1\bar{2}0$] (A) and 3#‐($0\bar{2}\bar{5}$)/[$5\bar{5}2$] (B) substrate orientations. Error bars indicate the standard deviation coming from 20 repeatedly recorded data points in the same sample. As demonstrated in Text S3, the increased Laue spot number in synchrotron diffraction can be regarded as an indicator of SG formation. (C) Pearson correlation coefficient between FWHM and spots number of three sets of duplicate data for sample 3#. (D,E) Laue patterns of representative layers corresponding to 1#‐(001)/[$1\bar{2}0$] (D) and 3#‐($0\bar{2}\bar{5}$)/[$5\bar{5}2$] (E) substrate orientations. (Right) Evolution of integrated diffraction curves, presented as intensity versus azimuth angle (χ). As demonstrated in Text S2, the strain gradient has a negligible contribution to the observed diffraction broadening.

Based on Laue diffraction results (Figure 2; Figure S1), we observed that the deposited layers corresponding to two of the four substrate orientations (1# and 2#) exhibited quasi‐SX structures. Peak broadening in the χ direction characterizes the local orientation distribution [26]. A significant accumulation of crystal defects, such as dislocations and strain gradient, was observed, as indicated by the increasing full width at half maximum (FWHM) (Figure 2A; Figure S1A). The dynamic evolution of rotation and FWHM during single‐layer L‐DED is shown in Figure 1B,C, respectively. Similar trends are also observed in other layer processes. Given the limitations of strain gradients in causing peak broadening beyond one degree, the peak broadening was primarily attributed to the presence of dislocations, as associated with geometrically necessary dislocations (GNDs) [22]. While the diffraction intensity of the first five layers showed a remarkable decrease, as the layer number grew, the intensity gradually dispersed, with clear splitting of diffraction spots (Figure 2D; Movies S1 and S2). This suggests a transformation from GNDs to geometrically necessary boundaries (GNBs), which led to the formation of sub‐grains [27, 28]. In contrast, the layers with the other two orientations (3# and 4#) showed the presence of an extensive number of columnar SGs. The FWHM for both orientations initially increased and then decreased by the tenth layer (Figure 2A; Figure S1B). As the FWHM of SX with substrate orientation decreased, the spot number remained high, indicating the formation of a large number of SGs. From the original diffraction patterns, the diffraction spots of SX did not split, while SG spots became more dominant by the tenth layer (Figure 2E; Movies S3 and S4). To quantitatively measure the correlation between diffraction broadening and SGs, we calculated the Pearson correlation coefficient of three sets of duplicate data for sample 3#, which shows a moderate correlation between them (Figure 2C). Besides, for all substrate orientations, the rotation of crystal orientation at an increased deposition layer was also observed, which was indicated by the shift of spots (Figure 2D,E).

Keyholes and pores are common macroscopic defects in AM that not only induce recoil pressure and Marangoni effects—thereby disturbing local heat flow—but also significantly influence grain growth and grain boundary evolution [15, 29]. For example, they can disrupt epitaxial grain growth or promote heterogeneous nucleation, both of which contribute to the formation of SGs [30]. To unveil whether SGs could originate from such defects, we conducted operando X‐ray imaging experiments during the L‐DED process at the BF beamline of HEPS. The morphology of the melt pool, melt track, and surface during the deposition of the third to fourth layers is shown in Figure 1D and Figure S2. Throughout the printing process and in the previously deposited layers, no keyholes or pore‐related defects were observed, owing to the use of a conduction‐mode laser strategy, which effectively suppresses the formation of keyholes and associated pores [14]. Therefore, we can exclude keyhole‐induced SG formation in this case. It is worth noting that randomly oriented, unmelted powder particles were present on the side and top surfaces. These can generate weak spurious diffraction signals during operando Laue measurements, as shown in Figure 2D.

Overall, based on the in situ data, we found that a common feature of SX growth under different substrate orientations is the generation of a large number of dislocations, with defects gradually accumulating as the number of deposited layers increases. However, a key distinction lies in the response to this crystal defect accumulation: for substrate orientations 1# and 2#, it leads to the formation of sub‐grains, whereas for orientations 3# and 4#, a significant number of SGs are formed with a decrease in FWHM of the SX peaks. These intriguing findings suggest a potential causal relationship between SG formation and defect accumulation—under specific orientations and stress states, the aggregation and migration of dislocations may promote local crystal rotation or even grain reconstruction, ultimately resulting in the formation of SGs.

### 2.2 Ex Situ Microstructure Analysis

**FIGURE 3:**
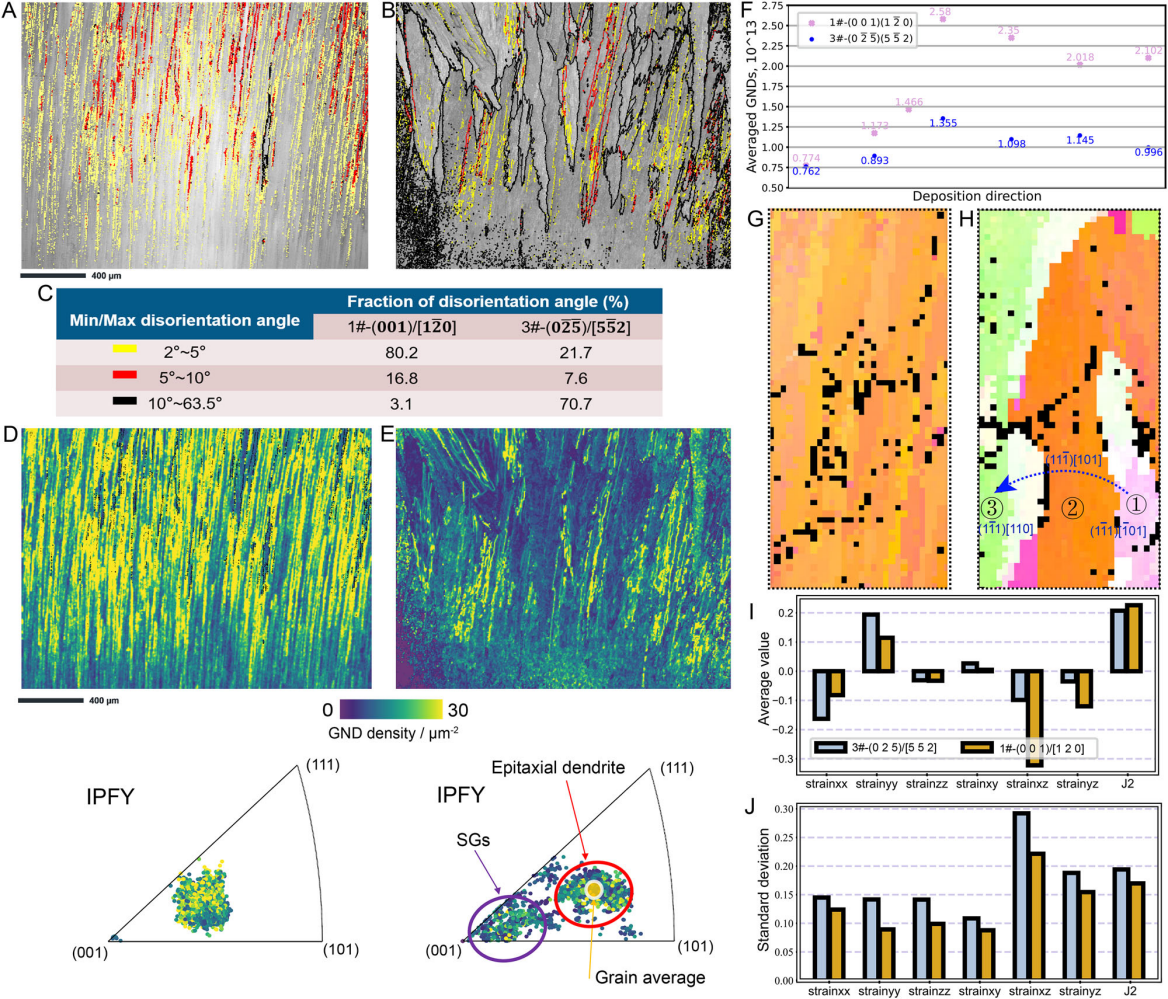
As‐printed microstructures and deformation analysis. (A,B), Grain boundary distribution maps corresponding to 1#‐(001)/[$1\bar{2}0$] (A) and 3#‐($0\bar{2}\bar{5}$)/[$5\bar{5}2$] (B) substrate orientations. The direction (or color) and size of the square diagram represent the orientation of the grains and the size of the region, respectively. (C) Statistical analysis of grain boundary disorientation angles, as shown in (A) and (B). (D,E) Distribution maps (Top) and IPF maps (Bottom) of GNDs corresponding to A,B. The color of the spots in IPF maps indicates the GND content. (F) Statistical average dislocation variation with printing height. The selected regions are shown in Figure S5a,c. (G–J) Residual deformation analysis determined by µLaue diffraction. Orientation map corresponding to 1#‐(001)/[$1\bar{2}0$] (G) and 3#‐($0\bar{2}\bar{5}$)/[$5\bar{5}2$] (H) substrate orientations and dislocation slip system analysis. Black pixels in the IPF represent unindexed data points. Statistical analysis of elastic deformation derived from Figure S6: Average value (I) and standard deviation (J) of the strain tensor components and J2. The sample sizes of Figure 3I,J are 2356.

As shown in Figure 2, the L‐DED process induces significant deformation and dislocation generation due to high shear strain, regardless of substrate orientation. These defects, however, play a crucial role in the formation of SGs. To statistically evaluate microstructural deformation and dislocation behavior at the macroscale, ex situ characterizations were performed using scanning electron microscopy (SEM) and electron backscatter diffraction (EBSD). The results are presented in Figure 3 and Figures S3–S5. For substrate orientations 1# and 2#, the as‐deposited grains exhibited elongated columnar morphologies with orientation continuity from the substrate to the top surface, indicating that epitaxial dendrites largely inherited the substrate crystallographic orientation (Figures S3A,B and S4). In contrast, for orientations 3# and 4#, although the initially deposited layers displayed epitaxial growth similar to that in orientations 1# and 2#, and the middle regions maintained morphology similar to the substrate, a transition occurred in the upper regions, where columnar SGs became dominant (Figures S3C,D and S4). Notably, the formation of columnar SGs did not initiate from the beginning of the deposition process.

**FIGURE 4:**
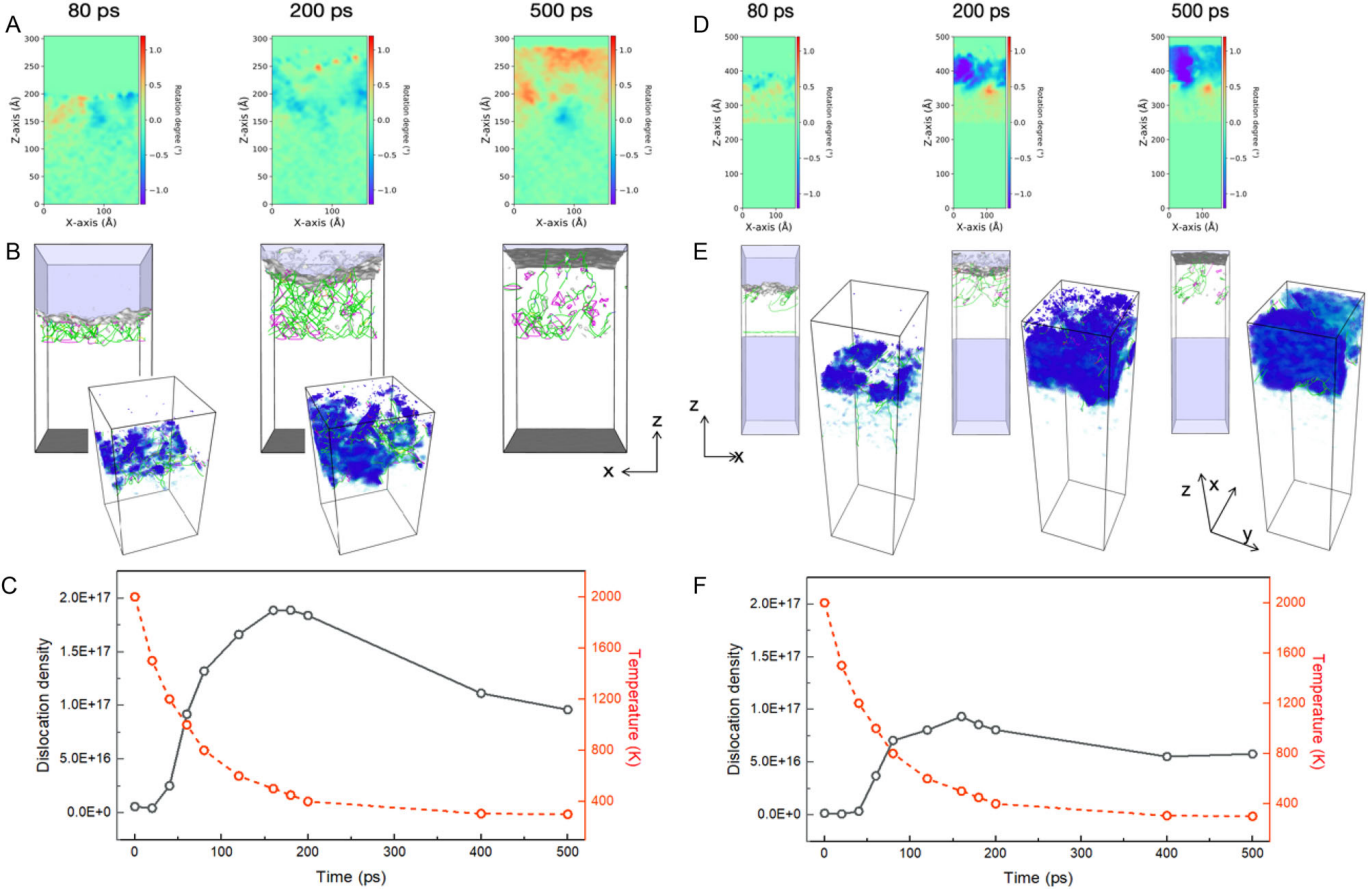
Molecular dynamics simulation of orientation and dislocation evolution during solidification. (A–C) Evolution behavior of orientation (A) and dislocation (B), and evolution of dislocation density over time (C) of the 1#‐(001)/[12 ®0] orientation. (D–F) Evolution behavior of orientation (D) and dislocation (E), and evolution of dislocation density over time (F) of the 3#‐($0\bar{2}\bar{5}$])/[$5\bar{5}2$] orientation.

A comparative analysis of grain boundaries (GBs) and GNDs for substrate orientations 1# and 3# is shown in Figure 3, while results for orientations 2# and 4# are provided in Figure S5. As illustrated in Figure 3A–C, low‐angle grain boundaries (LAGBs, misorientation < 10°) [31, 32] dominate in substrate orientation 1#, whereas high‐angle grain boundaries (HAGBs, misorientation > 10°) are prevalent in orientation 3#. This difference in GB types is attributed to the distinct migration behaviors of GNDs. As shown in Figure 3D and Figure S5D, orientations 1# and 2# exhibit a large number of LAGBs, where GNDs are retained within the grain interior rather than migrating to the boundaries to form HAGBs. In contrast, for orientations 3# (Figure 3E) and 4# (Figure S5E), GNDs undergo aggregation and rearrangement, migrating from the grain interior to the boundaries. This process results in the formation of numerous HAGBs and a corresponding reduction of GNDs within the SGs. Combining these observations with in situ Laue diffraction results, we infer that plastic deformation and dislocation activity may drive subgrain rotation, which ultimately leads to the formation of SGs.

Based on the EBSD analysis, dislocation motion and SG formation inevitably influence the local deformation field. To investigate the spatial distribution of deformation and its relationship with SG development, synchrotron‐based µLaue diffraction experiments were performed (Figure S6; see Materials and Methods section for details), which can supply information about elastic and plastic deformation [33, 34]. The degree of elastic deformation under different substrate orientations was quantified by calculating the second invariant (J_2_) of the deviatoric strain tensor, as shown in Figure 3G–J. Substrate orientation 1# exhibited higher average strain and larger standard deviation at the top of the deposited region compared to orientation 3#, which can be attributed to heterogeneity in the newly deposited layers that reduces the local yield strength. In these regions, deformation is accommodated through the formation of defects such as subgrain boundaries, resulting in peak broadening and splitting in Laue diffraction patterns (Figure 2D). However, the ability of LAGBs to accommodate deformation is limited, leading to significant residual strain that is unevenly distributed throughout the microstructure. In contrast, the strain in orientation 3# was lower and more uniformly distributed. Nevertheless, thermomechanical interactions among adjacent SGs induced localized compressive or tensile strains in different grains (Figure S6B), accounting for the observed intergranular strain heterogeneity.

Beyond elastic deformation, plastic deformation was also identified within SGs. By comparing the directions of experimentally observed peak broadening in Laue patterns with the simulated tensile directions of the twelve {111}<1$\bar{1}$0> slip systems in face‐centered cubic (FCC) Ni‐based superalloys (Figure S7), three activated slip systems were identified within individual SGs (Figure 3H). The variation in activated slip systems depends on local shear stress conditions, and the activation of different slip systems highlights the highly complex and inhomogeneous nature of the stress field. These results further demonstrate that significant deformation and dislocation motion occur during the L‐DED process, which facilitates subgrain rotation, ultimately promoting the formation of SGs.

### 2.3 Mechanism of Stray Grains Generation During L‐DED

Both in situ and ex situ results highlight two key factors influencing SG formation during the SX L‐DED process: the sensitivity to substrate orientation and the dependence on deformation accumulation. If the formation of columnar SGs were governed by the Columnar‐to‐Equiaxed Transition (CET), as predicted by conventional SG theories like CET, which rely solely on the thermal gradient [35, 7], no significant difference in SG content would be observed across different substrate orientations; similarly, little variation would be expected with different numbers of deposited layers. These findings indicate that deformation plays a pivotal role in driving SG formation, and that dislocation release pathways vary depending on the substrate orientation. The systematic confirmation of complex dislocation evolution during AM of 316L stainless steel by Gao et al. [20] using in situ XRD omitted the study of these phenomena in SX. To further elucidate the dynamic evolution of dislocations and their influence on SG formation under different substrate orientations during the L‐DED process, we conducted molecular dynamics (MD) simulations, guided by the combined insights from the aforementioned in situ and ex situ characterizations. A detailed comparison with the classic constitutional supercooling mechanism is demonstrated in Text S6.

Figure S8 illustrates the atomic evolution during solidification for substrate orientations 1# and 3#. For orientation 1#, the epitaxial plane corresponds to the (001) plane of the FCC structure, which possesses the highest symmetry and a tightly packed, ordered atomic arrangement (Figure S8A,B). The (001) plane also exhibits a higher atomic diffusion coefficient (Figure S8E), indicating more active atomic mobility along this crystallographic direction. Since the Gibbs free energy for homogeneous nucleation is higher than that for heterogeneous nucleation [36], grains with the <100> growth direction preferentially undergo epitaxial growth from the partially melted substrate to reduce solidification barriers. Under these conditions, deposited atoms can readily diffuse along the substrate's (001) orientation and occupy the energetically favorable lattice sites, promoting epitaxial alignment with the substrate crystal. Moreover, this orientation exhibits relatively low and uniformly distributed mismatch stress (Figure S8F), resulting in a lower interfacial energy (Figure S8G), which further favors the stability of the epitaxial growth front. Consequently, the epitaxial layer on the (001)‐oriented substrate maintains a low orientation deviation (∼0.5°, Figure 4A).

During the initial stage of solidification, the newly solidified grains exhibit relatively low yield strength due to their elevated temperature [31, 37]. The presence of steep thermal gradients induces plastic deformation, leading to the formation of dislocations (Figure 4B) and other crystal defects to relieve interfacial stresses [38, 39]. These high‐density dislocations dissipate by promoting the formation of LAGBs, which account for up to 90% of the observed boundaries (Figure 3A,C; Figures S5A,C, Figure 4C). The development of LAGBs helps accommodate internal stress fields within the crystal and impedes further dislocation motion [40], thereby suppressing subgrain rotation and preventing the transition to HAGBs, thus stabilizing the SX structure. However, due to their low GB energy and high dislocation energy, LAGBs have a limited ability to accommodate large‐scale deformation [41, 42, 43]. As a result, a substantial number of GNDs remain uniformly distributed within the grains (Figure 3D; Figure S5D), leading to significant residual elastic strains (Figure 3I).

In contrast to orientation 1#, where epitaxial growth is promoted by favorable lattice matching and effective dislocation accommodation through LAGBs, orientation 3# presents a markedly different solidification behavior. Here, the epitaxial plane corresponds to the (025) crystallographic plane, which lacks high symmetry and exhibits a less compact and ordered atomic packing compared to the (001) plane. After atomic relaxation, this orientation undergoes substantial surface lattice distortion (Figure S8C,D), increasing the instability of the solid–liquid interface. Additionally, a lower diffusion coefficient along the (025) plane (Figure S8E) indicates constrained atomic mobility, which prevents atoms from efficiently reaching low‐energy lattice sites. As solidification proceeds, these distortions give rise to significant lattice mismatch at the interface. The resultant mismatch stress is unevenly distributed, with some regions experiencing excessive strain (Figure S8F), which in turn leads to higher interfacial energy (Figure S8H). This accumulation of interfacial distortion gradually misaligns the growing epitaxial layer from the substrate, promoting subgrain formation with increasing misorientation (Figure 4D).

Moreover, the dislocation structure under this orientation is spatially heterogeneous (Figure 3B; Figure S5B, and Figure 4E), resulting in inefficient local stress relaxation and excessive residual stress in certain regions (Figure S6B). During solidification, these stresses drive the early‐stage aggregation and rearrangement of GNDs. However, the relatively low dislocation density and limited dislocation content along subgrain boundaries hinder the material's ability to accommodate strain and prevent subgrain rotation (Figure 4D,E). These factors lead to the movement and rearrangement of GNDs formation of HAGBs as subgrains rotate, accompanied by the consumption of dislocations (Figure 3B,C; Figure S5B,C). Once formed, SGs become largely free of dislocations which mainly distribute at GBs (Figure 3E; Figures S5E and S4E), and the total dislocation density decreases (Figure 4F). Potentially, the formation of HAGBs can be driven by shear‐coupled grain boundary migration mediated by the motion of grain boundary disconnections [44], dislocations absorbed at newly formed HAGBs or recovery/recrystallization‐like processes [45]. We speculate that the intermediate morphology evolution of dendrites during solidification under the dislocation‐mediated heterogeneous deformation is similar to the phenomenon of dendrite fragmentation observed by Wang et al. [46].

As deposition continues, competitively misoriented grains are gradually eliminated, while favorably oriented grains persist, leading to the formation of columnar SGs and a complete reorientation of the epitaxial structure. Plastic deformation also continues after columnar SG formation, activating distinct slip systems in different grains (Figure 3H). This process helps to further lower the free energy of the solidified structure and to accommodate the residual stress field (Figure 3I).

Collectively, these findings highlight that favorable substrate orientations enable uniform, high‐density dislocation networks that promote strain accommodation through LAGBs and suppress SG formation. In contrast, less favorable orientations result in heterogeneous dislocation distribution and insufficient strain coordination, thereby promoting subgrain rotation and the formation of columnar SGs.

## 3 Discussion

### 3.1 Evolution of GND and J2 in Multi‐Layer AM Process

Given the limited spatial and temporal scales in MD simulations, which hinder fully capturing the real multi‐physics and multi‐layer L‐DED process, we further developed a high‐fidelity multi‐physics model integrating melt pool dynamics and crystal plasticity in meso‐scale, to investigate the evolution of dislocation density during the printing of Ni‐based SX superalloys with different substrate orientations. These simulations were employed to be compared with experimental observations and to complement the MD results.

**FIGURE 5:**
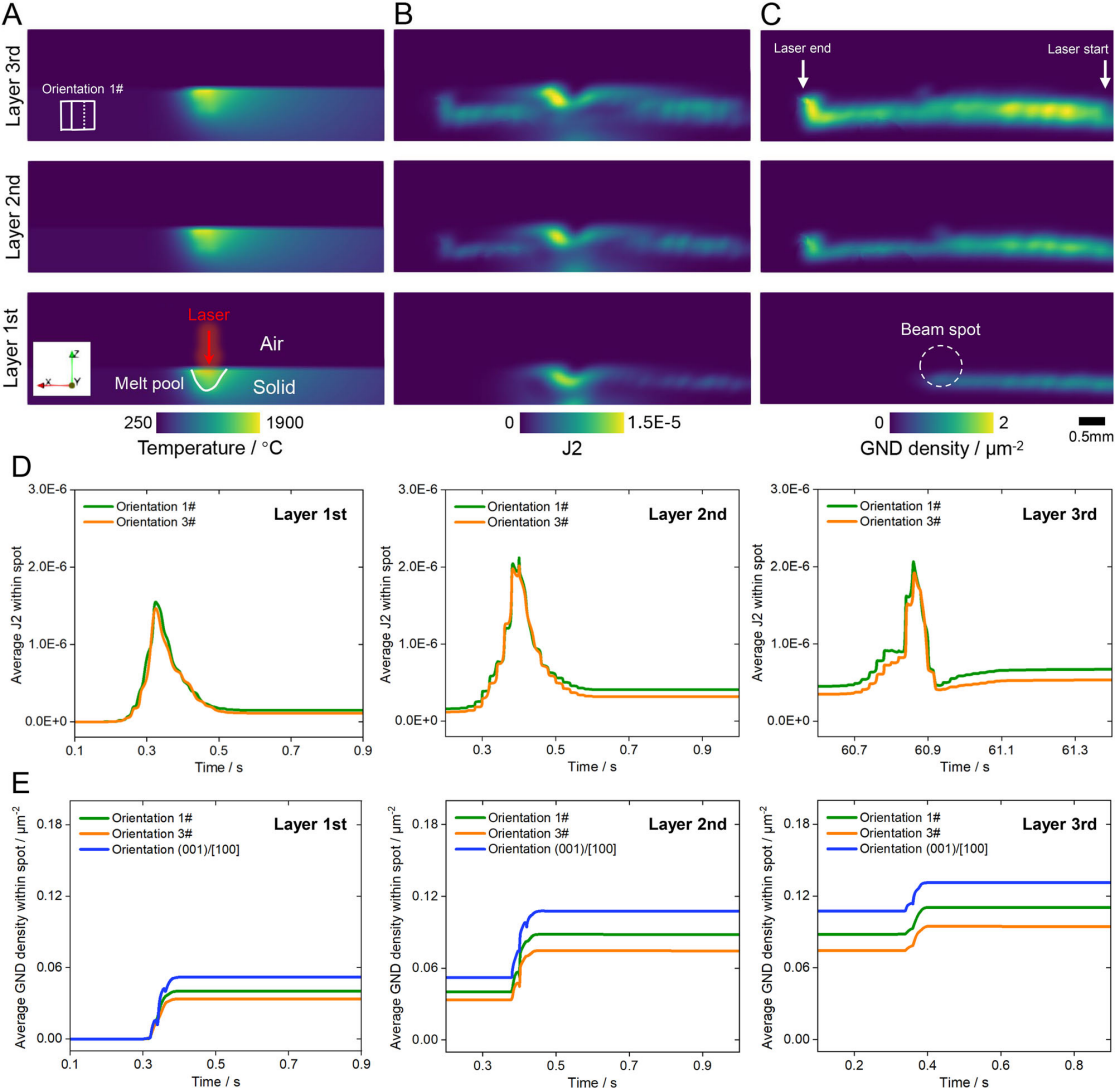
Evolution of temperature, J2, and GNDs during the L‐DED process of Nickel‐based SX superalloy, as simulated by multi‐physics modeling. (A–C) The patterns of temperature (A), J2 (B), and GNDs (C) during the L‐DED process at the substrate with orientations 1#. The temperature distributions provide information on solid/liquid phases and air, where the temperature gradient and thermal stress are the driving forces of plastic deformation. (D,E) The evolution of J2 (D) and GND densities (E), which are averaged within the area of the beam spot or illuminated region during three‐layer deposition for orientations 1# and 3# and additional orientation (001)/[100].

Figure 5A–C present the time‐resolved evolution of temperature, J2 (the second invariant of the strain deviator tensor) and GND density during three‐layer deposition for orientations 1# and 3#, with the whole dynamic evolutions provided in Supplementary Movies S5–S7. Driven by thermal stress, both J2 and GND accumulate along the scanning path, with their intensities amplified as the number of deposited layers increases. Due to the keyhole fluctuation, both J2 and GND exhibit notably non‐uniform distribution along the scanning path. As evidenced by the J2 mapping (Figure 5D), the high temperature gradient and thermal stress in the L‐DED process produce a more significant residual deformation‐induced residual elastic strain in orientation 1# as the laser has passed (Figures 5D and 2I), consistent with ex situ µLaue diffraction measurements (Figure 3F,I; Figure S6). Both indicate that the substrate orientation influences the degree of plastic deformation, under the identical temperature history and thermal stress load.

Furthermore, both the multi‐physics simulations and EBSD results indicate that orientation 1# exhibits a higher GND density than orientation 3# (Figures 5E and 3F), with the contrast between 1# and 3# becoming more pronounced as the deposition layer increases, which can be validated using the statistical results of multi‐layer EBSD mapping in Figure S17C,D. Despite GND density, orientation 1# similarly produces higher statistically stored dislocation (SSD) density throughout the L‐DED printing process (Figure S15), compared with orientation #3. This difference in total dislocation density between orientation #1 and #3 aligns well with the MD simulation results (Figure 4C,F), implying that the nucleation of SGs does not rely on the dislocation magnitude, whereas the dislocation Gini coefficient characterizing the scatter degree can provide better prediction. Therefore, the integration of ex situ characterization, in situ measurements, atomistic simulations, and macro‐scale modeling provides a consistent and cross‐validated understanding of the underlying mechanism of columnar SG formation during the L‐DED process.

### 3.2 Principle for SG Suppression

Cracking represents a critical forming defect in additively manufactured Ni‐based SX superalloys, severely compromising the mechanical properties and service reliability of components. In recent years, numerous in situ synchrotron radiation imaging studies have been conducted to elucidate the mechanisms underlying crack initiation and propagation during the additive manufacturing process [47, 48, 49]. The presence of SGs introduces high‐angle grain boundaries, which tend to act as sites for stress concentration under the high thermal gradients and solidification rates characteristic of additive manufacturing, thereby facilitating intergranular crack formation [50]. In this study, as shown in Figure S9, it was observed that cracks preferentially initiated and propagated along SG boundaries in specific orientations (e.g., 3# and 4#). In contrast, no cracking occurred during the printing process in orientations where SGs were absent (e.g., 1# and 2#). Hence, suppressing the formation of SGs emerges as an effective strategy to mitigate cracking at its source. Based on in situ Laue diffraction results, we further demonstrated that the formation of SGs is highly dependent on the substrate orientation. Given the virtually infinite combinations of substrate orientations and process parameters, it is highly impractical to rely solely on empirical trial‐and‐error approaches to identify optimal conditions for SX growth. Therefore, establishing a generalizable principle for SG suppression is essential for accelerating the design and optimization of printing parameters.

Considering the underlying mechanism of SG formation—the inhomogeneous distribution of dislocations inducing subgrain rotation—we propose that selecting substrate orientations with a lower Gini coefficient of dislocation distribution can effectively mitigate SG formation. The Gini coefficient here quantifies the uniformity of dislocation distribution, with lower values indicating more homogeneous dislocation distributions. Thus, the Gini coefficient provides a qualitative metric to assess the likelihood of SG formation under different crystallographic orientations.

To validate this principle, we conducted MD simulations to compute the interfacial energy and dislocation‐based Gini coefficients for various substrate orientations, as summarized in Table S2. The results show that orientations 1# and 2#, which did not produce SGs, exhibit relatively low Gini coefficients (0.169–0.245), whereas orientation 3#, which generated SGs, has a significantly higher Gini coefficient (0.38). To further test the robustness of this proposed criterion, we identified a new crystallographic orientation—(001)/[100]—via MD simulations, which exhibits an even lower Gini coefficient of 0.142, smaller than that of orientation 1#. According to the proposed suppression principle, this orientation should also inhibit SG formation under identical process parameters, thereby serving as a predictive validation of the criterion.

To further investigate the solidification behavior under the proposed orientation, we conducted in situ Laue diffraction experiments during the L‐DED process at the BSRF‐3W1 beamline, supplemented by ex situ EBSD characterization. A 17 × 17 inch dynamic flat‐panel detector (Mercu 1717HS, Iray) was employed to capture rich diffraction patterns. As shown in Figure S10A–D and g, h, the deposited layers largely retained the crystallographic orientation of the substrate while also exhibiting defect structures similar to those observed in Orientations #1 and #2. With increasing layer number, the diffraction intensity significantly weakened and became more diffuse, accompanied by evident spot splitting—indicative of the formation of GNDs and grain/subgrain boundaries GNBs.

In contrast to Orientations #1 and #2, the Laue spots under this orientation displayed smaller positional shifts, even during the reciprocal laser scanning process. The spot positions remained closely aligned with those of the substrate, indicating suppressed crystal rotation during deposition (Figure S10E,F). These observations were further corroborated by ex situ EBSD results (Figure S10i).

In summary, this set of experiments not only validates the proposed SG suppression principle but also identifies a promising orientation—(001)/[100]—based on this principle and under the current processing parameters. This orientation effectively inhibits SG formation while minimizing high‐angle lattice rotation, offering a favorable solution for SX additive manufacturing.

## 4 Conclusion

We systematically revealed the mechanism of SG formation during L‐DED of SX Ni‐based superalloys by integrating in situ synchrotron X‐ray imaging (HEPS), Laue diffraction (BSRF), ex situ EBSD/SEM/µLaue, MD simulations, and coupled thermal–fluid–crystal plasticity modeling. Imaging confirmed conduction‐mode melting without keyhole porosity, while diffraction and characterization showed that high‐symmetry substrate orientations maintained quasi‐SX structures through LAGBs accommodating deformation, whereas low‐symmetry orientations promoted inhomogeneous dislocation accumulation and columnar SGs. Simulations validated that plastic deformation, rather than constitutional undercooling, dominates SG evolution. We introduce the dislocation Gini coefficient as a quantitative predictor of SG suppression, with (001)/[100] orientation exhibiting the lowest coefficient and absence of SGs. This multiscale framework not only clarifies the causal role of dislocation distribution in SG formation but also offers a practical criterion for selecting substrate orientations and optimizing process parameters, paving the way toward reliable additive manufacturing of SG‐free SX alloys.

## 5 Materials and Methods

### 5.1 Materials

The samples consisted of nickel‐based superalloy bare plates and powder samples. The chemical composition of the SX nickel‐based superalloy sample is given in Table S3. The powder particle size ranges from 15 to 53 µm, with chemical composition identical to that of the substrate (as shown in Table S3). The crystal structure of nickel‐based superalloy is FCC γ phase. The SX bare plates with different orientations, were cut from a casting SX in different directions using electro‐discharge machining, with nominal dimensions of 15 mm (X) × 0.5 mm (Y) × 10 mm (Z). Four substrate orientations are listed in Table S1. High‐symmetry orientation refers to the configuration where the crystallographic plane of the substrate normal to the direction of laser incidence is a high‐symmetry plane, such as (001).

### 5.2 In Situ Laue Diffraction, X‐ray Imaging and Data Analysis

The operando Laue diffraction platform for the L‐DED process was designed and built at 3W1 beamline at BSRF, as shown in Figure 1A. The probe ‘white’ X‐rays were generated from a superconducting wiggler with a broad band spectrum (Figure S11). A transmission geometry was used for the X‐ray diffraction measurements, with the X‐rays incident from the ‐Y direction. An SX tungsten pinhole placed in front of the sample provided a 250 µm × 250 µm square beam spot. The EIGER X 1 m detector (Dectris, Ltd.) was positioned 93.26 mm downstream of the sample and operated at a frame rate of 200 Hz with an exposure time of 5 ms per frame.

The four‐orientation operando Laue diffraction patterns, which varied with the number of deposited layers, are shown in Supplementary Movies S1–S4. Laue spot indexing was conducted by Lauetools [51]. The diffracted spots correspond to the γ phase of the SX nickel‐based superalloy. The results are shown in Figure S12. To quantitatively assess the broadening of the diffraction peaks, we used the Python‐based lmfit library to perform 2D Gaussian fitting on the diffraction patterns [52]. This enabled us to track the variation in peak broadening as the number of printed layers increased, as shown in Figure 2 and Figure S1.

Since the orientations of SGs differ from those of epitaxially grown grains, a higher SG content leads to an increased number of discrete Laue diffraction spots. Therefore, the number of Laue spots can, to some extent, be used as an indicator of SG content [22]. To determine the diffraction spot number, intensity, and area thresholds were set at 60 and 4, respectively.

In situ X‐ray imaging during the L‐DED process was conducted at the Test Beamline of the HEPS. This Wiggler‐based beamline provides X‐ray energies ranging from 10 to 300 keV, enabling in situ imaging of heavy material systems such as SX nickel‐based superalloys. In the coupled imaging system, a 100 µm LuAg:Ce scintillator was used in combination with a 5× objective lens and a FASTCAM SA‐Z high‐speed camera (Photron). The frame rate and exposure time of the camera were set to 1 kHz and 980 µs, respectively. The morphology of the newly deposited layer and the melt pool is shown in the insert of Figure 1A.

### 5.3 Laser‐Direct Energy Deposition Apparatus

The L‐DED device (Figure 1A) consists of a laser, a sample motion stage, a powder feeding system, a laser processing head, and a control system. The laser is a fiber‐output semiconductor laser (RFL‐A2000D, Wuxi Raycus Fiber Laser Technologies Co., Ltd) with a wavelength of 915 nm and a maximum power of 2000 W. The sample motion stage is used to hold the sample and allows for high‐precision 3D movement. The powder feeding system is equipped with a dual‐cylinder powder feeder, utilizing argon gas to transport powder materials, capable of simultaneously delivering two types of powder.

The laser processing head can move in three dimensions and delivers both the focused laser beam and the powder material to the printing area on the sample stage. The minimum size of the laser focus spot is 100 µm, with the laser and powder being coaxial. The powder feeding rate is adjusted by controlling the argon gas flow rate, which also serves as a shielding gas to prevent oxidation and defects in the sample.

The control system manages the entire setup, including laser power, movement of the sample stage (direction, distance, speed), powder feeding control, movement of the laser processing head (direction, distance, speed), and scanning strategies (single‐pass scan, multi‐pass unidirectional scan, multi‐pass multidirectional scan), among other parameters. All L‐DEDed samples were fabricated using a laser power of 238 W, a laser scanning speed of 800 mm/min, and a scanning length of 10 mm for a single track. The process strategy was reciprocal laser scanning.

### 5.4 Synchrotron µlaue Diffraction

The synchrotron µLaue diffraction experiments were performed at the BL03HB beamline of the Shanghai Synchrotron Radiation Facility (SSRF) using a reflection geometry. The polychromatic X‐rays had an energy range of 7 to 28 keV, with a beam spot size of 3 µm × 3 µm. A PILATUS3 S 2 m detector (Dectris, Ltd.) was used, with an exposure time of 0.5 s. The µLaue data were processed using Lauetoolsnn [53] software to determine the orientation and strain distribution (Figure S6). Laue diffraction patterns with streaked reflections, taken from different SGs, were analyzed using Lauetools [51] to obtain dislocation slip systems (Figure S7). All the 12 possible {111} <$1\bar{1}0$> dislocation slip systems of FCC Ni‐based superalloy were considered, and three SGs with different orientations were studied in this article.

### 5.5 Microstructure Characterization

A LEICA DMC 4500 optical microscope was adopted to characterize the microstructure of 20‐layer deposition samples, which were etched by a mixture solution of 100 mL HCl + 100 mL C_2_H_2_OH + 50 g CuCl_2_. A JEOL JSM7100 scanning electron microscope was used to conduct EBSD analysis (the step size was 3 µm). The collected EBSD data were processed using ATEX software to calculate orientation maps (Figure S5), misorientation angle, and GND (or Nye tensor) (Figure 3; Figure S5) [54], with the detailed algorithm provided in Text S2.

### 5.6 Molecular Dynamics Simulation

In this study, MD simulations were performed using the LAMMPS package to investigate the microstructural evolution during laser melting and directional solidification. The simulation system was based on FCC structure of nickel, with interatomic interactions described by the embedded atom method potential developed by Zhou [55], which accurately captures atomic behavior during melting, solidification, and dislocation evolution.

The simulation domain measured 100 nm × 100 nm × 400 nm and contained approximately 100 million atoms, ensuring both statistical relevance and the ability to resolve key microstructural features. Periodic boundary conditions were applied along the solidification direction (z‐axis), while free boundary conditions were imposed in the transverse directions (x and y) to emulate realistic boundary effects during epitaxial growth. Two substrate orientations were modeled: Orientation 1# as (001)/[12̅0] and Orientation 3# as (02̅5̅)/[55̅2], with directional solidification proceeding along the z‐direction.

To replicate the complex thermal field during laser additive manufacturing, a temperature profile was extracted from finite element simulations and scaled to the atomistic level. The temperature gradient was applied using a Langevin thermostat along the z‐axis, mimicking localized melting and directional solidification induced by laser heating. The initial temperature was set to 300 K. Gradual heating led to complete melting of the substrate region, followed by controlled cooling through a thermal gradient to achieve directional solidification of the molten metal. The time step was fixed at 1 fs to ensure temporal resolution and numerical stability.

Post‐processing analyses were conducted on atomic configurations at various simulation stages. Common neighbor analysis was used to identify lattice structures (FCC, HCP, and amorphous regions), allowing for visualization of stacking faults and subgrain formation. Local stress distributions were calculated from atomic stress tensors to examine the spatial variation of lattice mismatch stresses and their influence on orientation deviation. Additionally, the dislocation extraction algorithm was applied to trace dislocation nucleation, motion, and annihilation, and to quantify dislocation density and slip activity, thereby assessing their roles in stress relief and crystal structure stabilization. As the indicator of columnar SG formation, the Gini coefficient of dislocations at the solid‐liquid interface is derived from MD results, with a detailed algorithm provided in Text S4.

### 5.7 Thermal‐Fluid Flow Model

A multiphysics thermal flow model is used to simulate the molten pool flow during the L‐DED process. To increase the accuracy of the simulation, the powder particle size, velocity, and temperature distribution, and laser energy distribution above the substrate surface are simulated by multi‐phase powder streaming simulation model first [56]. These powder and laser features are then incorporated into the Multiphysics thermal‐fluid flow model to simulate the molten pool flow, where the heat transfer, liquid flow, particle inserting, Marangoni effect, etc., are further incorporated. In the thermal‐fluid flow model, the liquid is assumed to be incompressible Newtonian laminar flow and the mass, momentum, and energy conservation equations are given as [57, 58]:

$$\left\{\begin{array}{c} \rho \nabla \cdot \;\vec{v}=m_{p}\;\\ \rho \frac{\partial \vec{v}}{\partial t\;}+\rho \nabla \cdot \;\left(\vec{v}⊗\vec{v}\right)=-\nabla p+\nabla \cdot \left(\mu \nabla \vec{v}\right)+{\vec{f}}_{B}+\rho D\vec{v}+{\vec{f}}_{p}\\ \rho \frac{\partial I}{\partial t}+\rho \nabla \cdot \;\left(I\vec{v}\right)=\nabla \cdot \left(k\nabla T\right)+q+q_{p} \end{array}\right.$$

where ρ, μ, and *k* are the density, dynamic viscosity, and thermal conductivity of the material, respectively. *m_p_* is the mass increase by the powder particle. $\vec{v}$ and $\vec{g}$ are the velocity and gravitational acceleration vector. *p* is the pressure. $\overset{\to }{\underset{b}{f}}$ is the buoyancy force. *D* is the Darcy drag force coefficient [59], calculated by the Blake‐Kozeny model. $\overset{\to }{\underset{p}{f}}$ is the force exerted by the inserting powder particles. *I* and *T* is the specific enthalpy and temperature of the material. *q_p_* is the internal energy of the powder particles. *q* is the absorbed laser energy, which is calculated by a ray‐tracing method to track the laser reflection [58]. To capture the fluctuation of the molten pool surface, the volume of fraction method is applied:

$$\frac{\partial F}{\partial t}+\nabla \cdot \;\left(F\vec{v}\right)=0$$

where *F* is the volume fraction. Moreover, on the molten pool surface, the recoil pressure by the metal evaporation, Marangoni effect, and pressure by the surface tension are added as momentum boundary conditions, and given as:

$$\left\{\begin{array}{c} \;p_{n}=\sigma \kappa +P_{rec}\\ \;\tau_{t}=\sigma_{s}^{T}\;\left[\nabla T-\hat{n}\left(\nabla T\cdot \hat{n}\right)\right] \end{array}\right.$$

where *p_n_* and τ_*t*_ are the normal and tangential directional forces on the unit area. κ ad $\hat{n}$ are the curvature and unit normal vector on the free surface. σ and $\sigma_{s}^{T}$ are the temperature‐dependent surface tension and sensitivity of the surface tension coefficient. *P_rec_* is the recoil pressure by the metal evaporation [58]. Additionally, heat convection, radiation, and heat loss of evaporation are adopted for the thermal boundary on the free surface [58]. The parameters used for the simulations are listed in Tables S3 and S4. The results of the thermo‐fluid coupling simulations are presented in Movie S5.

### 5.8 Crystal Plasticity Model

Taking the time‐dependent temperature fields obtained from the thermal–fluid flow model as input, a crystal plasticity framework is employed to compute the thermal‐stress‐induced deformation and dislocation accumulation within the solid domain. The solid regions are assumed to be SX, sharing the same crystallographic orientation as the substrate, corresponding to the stage prior to SG formation.

The total deformation gradient **F** can be decomposed into elastic part **F_e_** and plastic part **F_p_**:

$$F=F_{e}\;\;F_{p}$$

in which the residual deformation **F_p0_** is taken as the initial condition of the plastic deformation gradient, to capture the effects of residual stresses. The Green–Lagrange elastic strain tensor is written as [60, 61]:

$$\varepsilon_{e}=\frac{1}{2}\left(F_{e}^{T}F_{e}-I\right)$$

The isotropic thermal expansion is employed to calculate the thermal stresses. Then, the 2^nd^ Piola‐Kirchhoff stress tensor is calculated as follows [62]:

$$\sigma =\left(C:\varepsilon_{e}-\alpha I\right)$$

where **C** is the elasticity tensor, and α is the thermal expansion coefficient.

To capture the gas and fluid regions within the simulation domain, we modify the stiffness and thermal expansion for those regions:

$$C^{\prime }=\xi C,\alpha^{\prime }=\xi \alpha$$

where *ξ* is a coefficient equal to 0.001.

The shear strain rates ${\dot{\gamma }}^{s}$ across all slip systems collectively contribute to the plastic strain rate:

$${\dot{\varepsilon }}_{p}={\dot{F}}_{p}\;{F_{p}}^{-1}=\sum_{s=1}^{N_{\mathrm{slip}}}\left(m^{s}⊗n^{s}\right){\dot{\gamma }}^{s}$$

where *N*_slip_ is the number of slip systems, **m*^s^*** is the unit vector along the slip direction, and **n*^s^*** is the unit vector normal to the slip plane.

The plastic flow rule proposed by Peirce et al. is employed to calculate the slip rate in each slip system [63, 64]:

$${\dot{\gamma }}^{s}={\dot{\gamma }}_{\mathrm{ref}}\;{\left(\frac{\left|\tau^{s}\right|}{\tau_{c}^{s}}\right)}^{n}\mathrm{sign}\left(\tau^{s}\right)$$

where $\tau_{c}^{s}$ is known as critical resolved shear stress (CRSS) [65], *n* is the exponent of the power law, and ${\dot{\gamma }}_{\mathrm{ref}}$ is the reference slip rate.

The $\tau_{c}^{s}$ in Equation [63] is written as [66]:

$$\tau_{c}^{s}=\tau_{0}^{s}+\varphi_{0}\;Gb\sqrt{\sum_{\alpha =1}^{N_{\mathrm{slip}}}\chi_{\alpha }^{s}\left(\left|\rho_{\mathrm{SSD}}^{\alpha }\right|+\left|\rho_{\mathrm{GNDe}}^{\alpha }\right|+\left|\rho_{\mathrm{GNDs}}^{\alpha }\right|\right)}$$

where $\rho_{\mathrm{GNDe}}^{\alpha }$ and $\rho_{\mathrm{GNDs}}^{\alpha }$ are the edge and screw components of GND density, $\rho_{\mathrm{SSD}}^{\alpha }$ is the immobile SSD density, φ_0_ is the pre‐factor of the Taylor hardening law and $\chi_{\alpha }^{s}$ is the latent hardening matrix, which captures the hardening interactions between different slip systems. Parameter *b* represents the norm of Burgers vector for the dislocation.

The SSD densities are written as [67]:

$${\dot{\rho }}_{\mathrm{SSD}}^{s}=\left(K\sqrt{\left|\rho_{\mathrm{SSD}}^{s}\right|+\left|\rho_{\mathrm{GNDe}}^{s}\right|+\left|\rho_{\mathrm{GNDs}}^{s}\right|}-2y_{c}\left|\rho_{\mathrm{SSD}}^{s}\right|\right)\frac{\left|{\dot{\gamma }}^{s}\right|}{b}$$

where *K* is a coefficient representing the accumulation rate and *y*_c_ is the critical annihilation distance for SSD, which measures the intensity of dislocation annihilation. The GNDs are incorporated to consider the extra strengthening effect induced by strain gradient [68]:

$$\left\{\begin{array}{c} \;\rho_{\mathrm{GNDe}}^{s}=-\frac{1}{b}\nabla \gamma^{s}\cdot n^{s}\\ \;\rho_{\mathrm{GNDs}}^{s}=\frac{1}{b}\nabla \gamma^{s}\cdot t^{s} \end{array}\right.$$

where **t** ^*s*^ = **n** ^*s*^  × **m** ^*s*^ is the direction of screw dislocation motion. In the subsequent simulation‐based analysis, the scalar SSD and GND are defined using the L2 norm:

$$\left\{\begin{array}{c} \left\|\rho_{\mathrm{GND}}\right\|=\sqrt{\sum_{s=1}^{N_{\mathrm{slip}}}\left({\left(\rho_{\mathrm{GNDe}}^{s}\right)}^{2}+{\left(\rho_{\mathrm{GNDs}}^{s}\right)}^{2}\right)}\\ \left\|\rho_{\mathrm{SSD}}\right\|=\sqrt{\sum_{s=1}^{N_{\mathrm{slip}}}{\left(\rho_{\mathrm{SSD}}^{s}\right)}^{2}} \end{array}\right.$$

The parameters used for the simulations are listed in Table S6. The results of the thermo‐fluid‐crystal plasticity coupling simulations are presented in Movies S6–S7. The above crystal‐plasticity model is applied to the solid SX domain. When the deformed solid transfers into liquid within the melt pool, all the previously accumulated SSD/GND and **F**_p_ are set to zero.

The temperature‐dependent CRSS and elastic properties were calibrated using the experimental results from the material datasheet for the Ni‐based SX superalloys [69]. The remaining parameters employed in the crystal‐plasticity simulations were adopted from our previous studies on the same type of Ni‐based superalloys, where they have been extensively validated against tensile stress‐strain, fatigue responses and creep curves over a wide range of temperatures [70, 65, 71]. The details of parameter calibration are presented in Table S6 and Figure S14.

## Conflicts of Interest

The authors declare no conflicts of interest.

## Supporting information




**Supporting file 1**: advs74236‐sup‐0001‐SuppMat.docx.

**Supporting file 2**: advs74236‐sup‐0002‐MoviesS1.avi.

**Supporting file 3**: advs74236‐sup‐0003‐MoviesS2.avi.

**Supporting file 4**: advs74236‐sup‐0004‐MoviesS3.avi.

**Supporting file 5**: advs74236‐sup‐0005‐MoviesS4.avi.

**Supporting file 6**: advs74236‐sup‐0006‐MoviesS5.mp4.

**Supporting file 7**: advs74236‐sup‐0007‐MoviesS6.mp4.

**Supporting file 8**: advs74236‐sup‐0008‐MoviesS7.mp4.

**Supporting file 9**: advs74236‐sup‐0009‐Movies.zip.

## Data Availability

The data that support the findings of this study are available from the corresponding author upon reasonable request.
