# Supplementary material for: Deformation‐Induced Formation of Stray Grains in Additive Manufacturing of Single Crystals
Source: Adv Sci (Weinh). 2026 Feb 15;13(25):e22704. doi: 10.1002/advs.202522704 (PMC13137851; doi:10.1002/advs.202522704)
Supplement: Supplementary file 1 — Supporting file 1: advs74236‐sup‐0001‐SuppMat.docx. [file ADVS-13-e22704-s004.docx]

Supplementary Materials for

**Deformation-Induced Formation of Stray Grains in Additive Manufacturing of Single Crystals**

Dongsheng. Zhang^1^*, Zixu. Guo ^2^*, Yuxiao. Li^1^, Lu. Wang^2^, Yu. Wu^3^, Darui. Sun^1^, Wentao. Yan^2^, Yuanyuan. Guo^4^, Han. Wang^3^, Wei. Liu^3^**, Ye. Tao^1^**, Bingbing. Zhang^1,5^**

^1^Institute of High Energy Physics, Chinese Academy of Sciences, Beijing, 100049, China.

^2^ Department of Mechanical Engineering, National University of Singapore, Singapore, 117575, Singapore.

^3^ 3D Printing Research and Engineering Technology Center, Beijing Institute of Aeronautical Materials, Beijing, 100095, China.

^4^ Science and Technology on Advanced High Temperature Structural Materials Laboratory, Beijing Institute of Aeronautical Materials, Beijing, 100095, China.

^5^ University of Chinese Academy of Sciences, Beijing, 100049, China.

* These authors contributed equally to this work.

**Corresponding author. Email: liuwei2011621@sina.com (W.L.), taoy@ihep.ac.cn (Y.T.), zhangbb@ihep.ac.cn (B.Z.).

**The PDF file includes:**

Supplementary Texts 1-6

Figures S1 to S18

Tables S1 to S6

**Other Supplementary Materials for this manuscript include the following:**

Movies S1 to S7

Supplementary Text 1

X-ray imaging analysis

Figure S2 illustrates the evolution of the melt pool, melt track, and surface morphology following the third-layer deposition, during the fourth-layer printing process, and after the completion of the fourth layer. During printing, the laser melts the substrate to form a melt pool, into which powder particles are injected and subsequently melted. As the melt pool progresses, its rear end rises due to the accumulation of molten material. The injection of room-temperature powder particles into the high-temperature, high-gradient melt pool induces localized quenching effects, resulting in transient thermal oscillations and a more complex temperature gradient field. As the laser scans, the melt pool solidifies at its trailing edge, forming a continuous melt track. During this process, partially melted powder adheres to the top and side surfaces of the melt track. While the powder on the upper surface can be remelted during subsequent layer deposition, the powder on the side surfaces is less likely to be re-exposed to the laser and thus tends to remain unmelted. These unmolten particles, with their random crystallographic orientations, contribute weak and diffuse signals in the in-situ Laue diffraction patterns (Figure 2D). Importantly, due to the use of a conduction-mode laser strategy, no keyhole formation or associated porosity was observed throughout the process. This rules out pore-induced crystalline disruption as a potential origin of stray diffraction signals. Therefore, the observed diffuse Laue signals are primarily attributed to residual sidewall powders rather than defects such as keyhole-induced porosity.

Synchrotron μlaue diffraction analysis

To obtain spatial deformation information of the printed samples, synchrotron-based μLaue diffraction experiments were performed. Figure S6 presents the inverse pole figures (IPFs) and strain tensor distributions at the top surface of the deposited layers for substrate orientations 1# and 3#. In these maps, black dots indicate regions where the experimental data could not be resolved. As shown in Figure S6A, the IPF along the experimental coordinate system reveals the presence of numerous subgrains at the top surface of orientation 1#, accompanied by a highly heterogeneous distribution of the strain tensor. In contrast, for orientation 3# (Figure S6B), a large number of columnar SGs are observed. While the strain fields within individual grains appear more uniform, thermal-mechanical interactions among adjacent SGs result in heterogeneous deformation—some grains experience compression while others undergo tension—leading to significant intergranular strain variation. To quantitatively assess the degree of elastic deformation under the two orientation conditions, we calculated the second invariant of the deviatoric strain tensor (J₂) based on the strain maps in Figure S6. Statistical analysis of the mean and standard deviation of the strain tensors within the characterized regions is summarized in Figure 3G-H.

Due to the inherently steep temperature gradients in additive manufacturing, not only elastic deformation but also plastic deformation can occur within SGs. We employed LaueTools to analyze Laue diffraction patterns collected from different SGs. By simulating the streaking directions corresponding to the 12 possible {111} <$1\bar{1}0$> slip systems in FCC Ni-based superalloys, as shown in Figure S7, we compared the simulated streak orientations with the experimentally observed broadening directions of diffraction peaks in Laue patterns. Based on this comparison, we successfully identified the activated slip systems in the three SGs shown in Figure 3H, which are: (1$\bar{1}$1)[$\bar{1}$01], (11$\bar{1}$)[101], and(1$\bar{1}$1)[110]


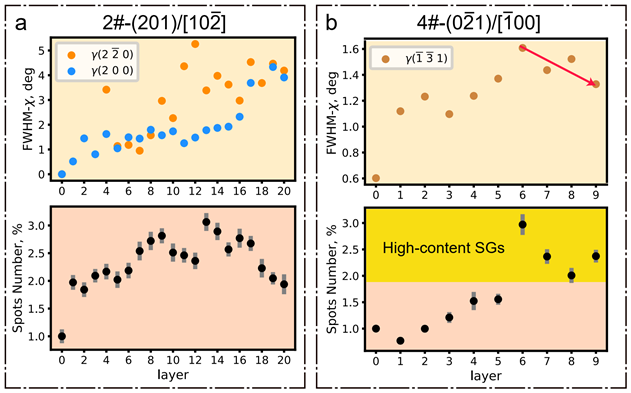


Figure S1.

Evolution of FWHM and spot number as a function of increasing printing layers during the L-DED process for 2#-($201$)/[$10\bar{2}$] (**A**) and 4#-($0\bar{2}1$)/[$\bar{1}00$] (**B**) substrate orientations.


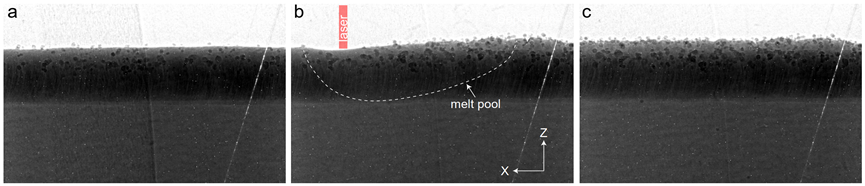


Figure S2.

**In situ synchrotron X-ray imaging melt pool quantification of L-DED nickel-based single crystal superalloy.** (**A**) The 3rd layer morphologies. (**B**) Melt pool during the 4th layer deposition. (**C**) The 4th layer morphologies. One pixel represents 4 microns.


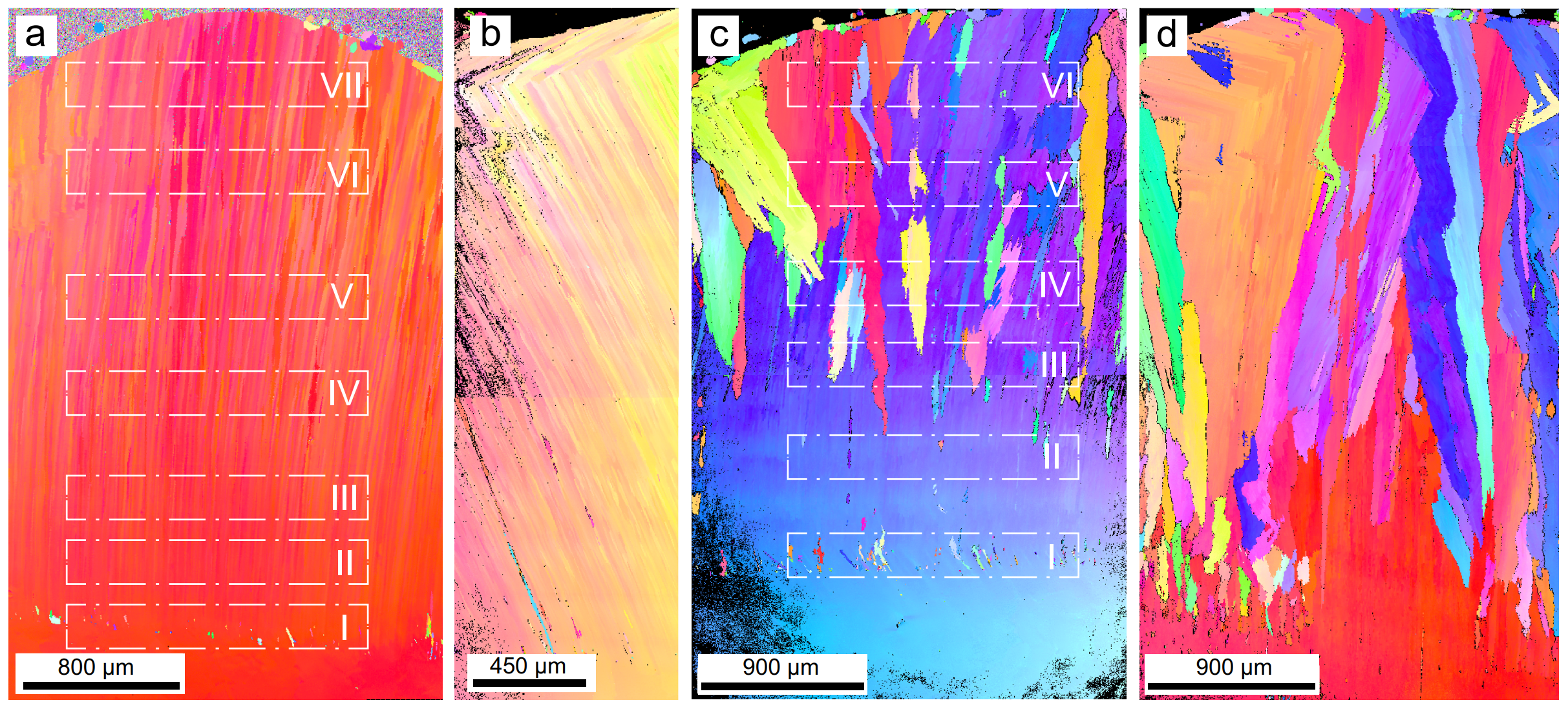


Figure S3.

**EBSD results of four-orientation L-DEDed samples**. Orientation maps (IPF-Z) corresponding to (**A**) 1#-($001$)/[$1\bar{2}0$], (**B**) 2#-($201$)/[$10\bar{2}$], (**C**) 3#-($0\bar{2}\bar{5}$)/[$5\bar{5}2$] and (**D**) 4#-($0\bar{2}1$)/[$\bar{1}00$] substrate orientations.


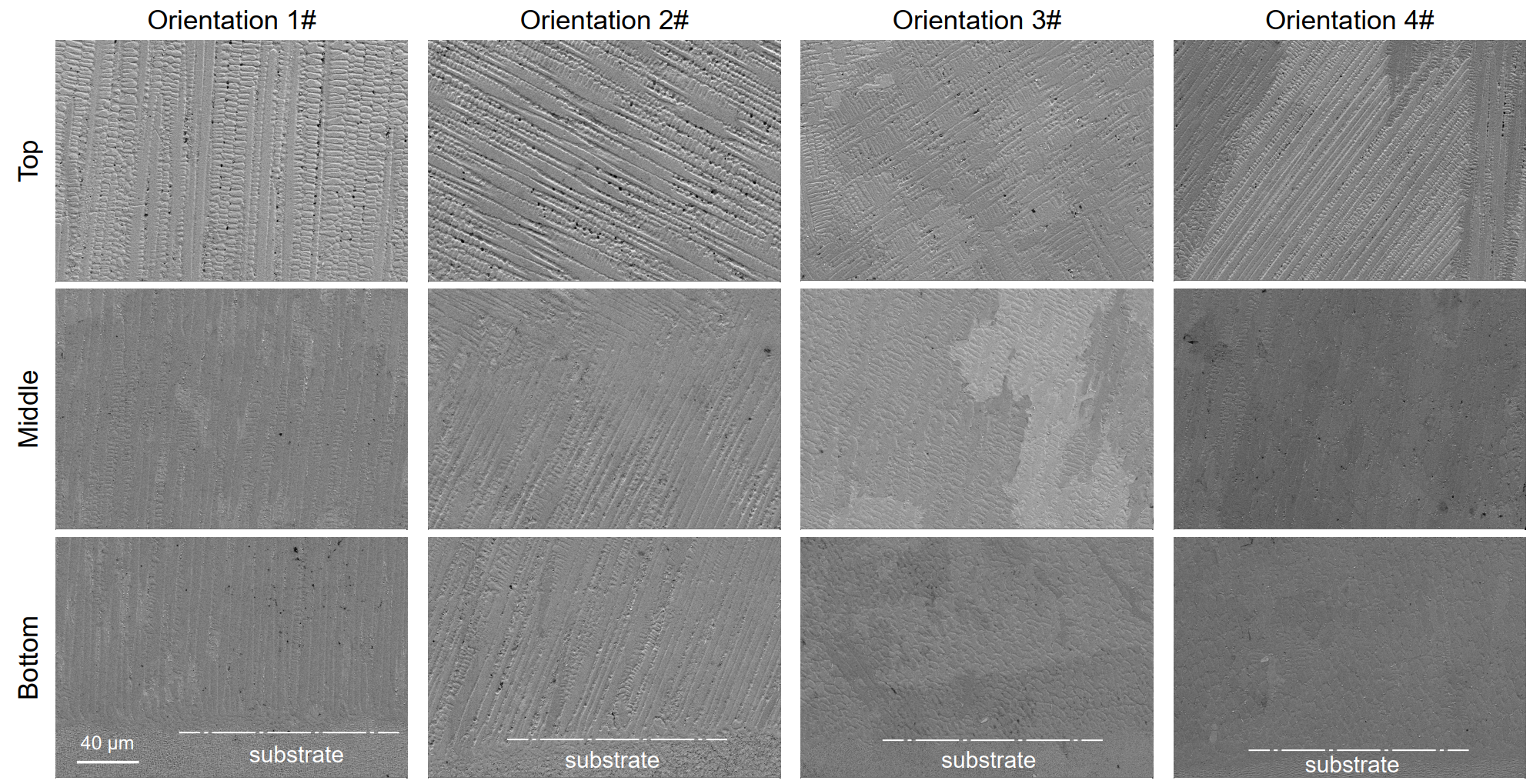


Figure S4.

**SEM images for the L-DEDed samples with four substrate orientations**. Above the substrates, significant dendrite structures within columnar grains can be observed, where the dendrite growth direction corresponds to the [001] orientation.


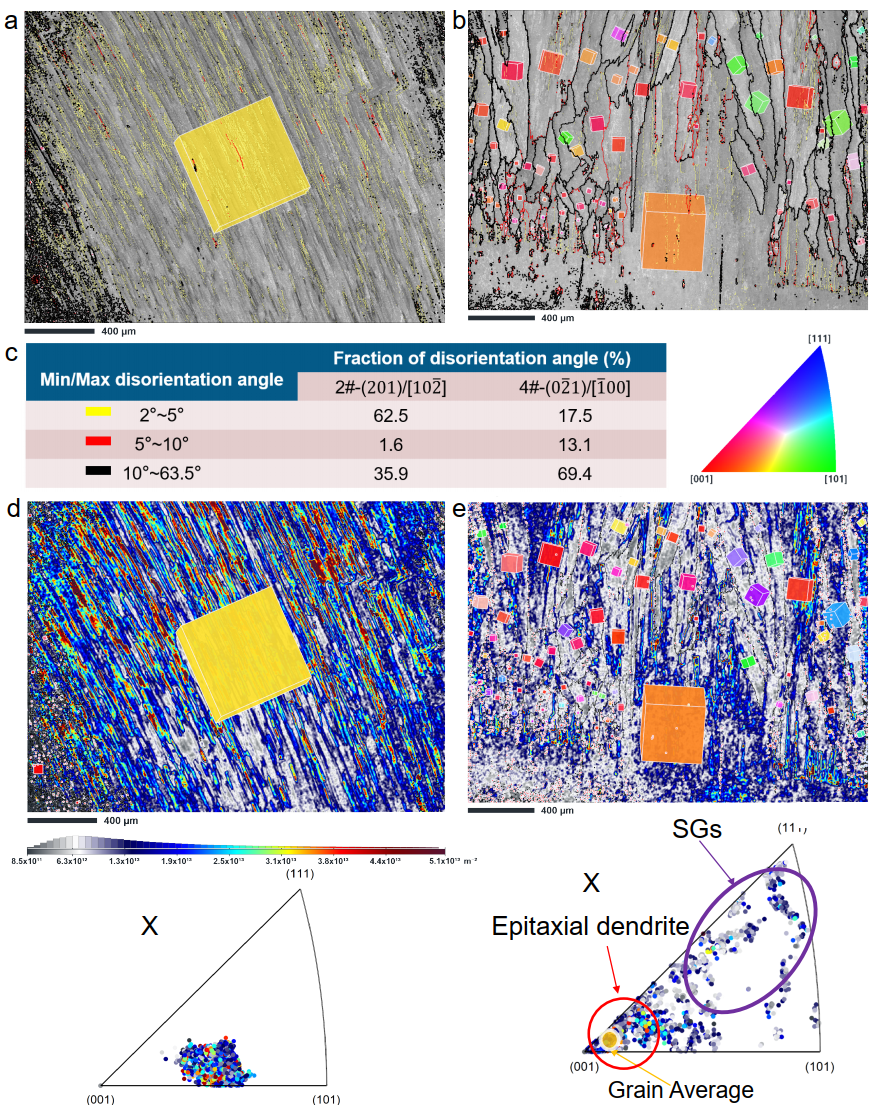


Figure S5.

**As-solidified microstructures of the 20-layer printed region.** (**A-B**) Grain boundary distribution maps corresponding to (A) 2#-($201$)/[$10\bar{2}$] and (B) 4#-($0\bar{2}1$)/[$\bar{1}00$]. The direction (or color) and size of the square diagram represent the orientation of the grains and the size of the region, respectively. (**C**) Statistical analysis of grain boundary disorientation angles, as shown in (A) and (B). (**D-E**) Distribution maps (Top) and IPF maps (Bottom) of GNDs corresponding to (A) and (B). The color of the spots in IPF maps indicates the GND content.


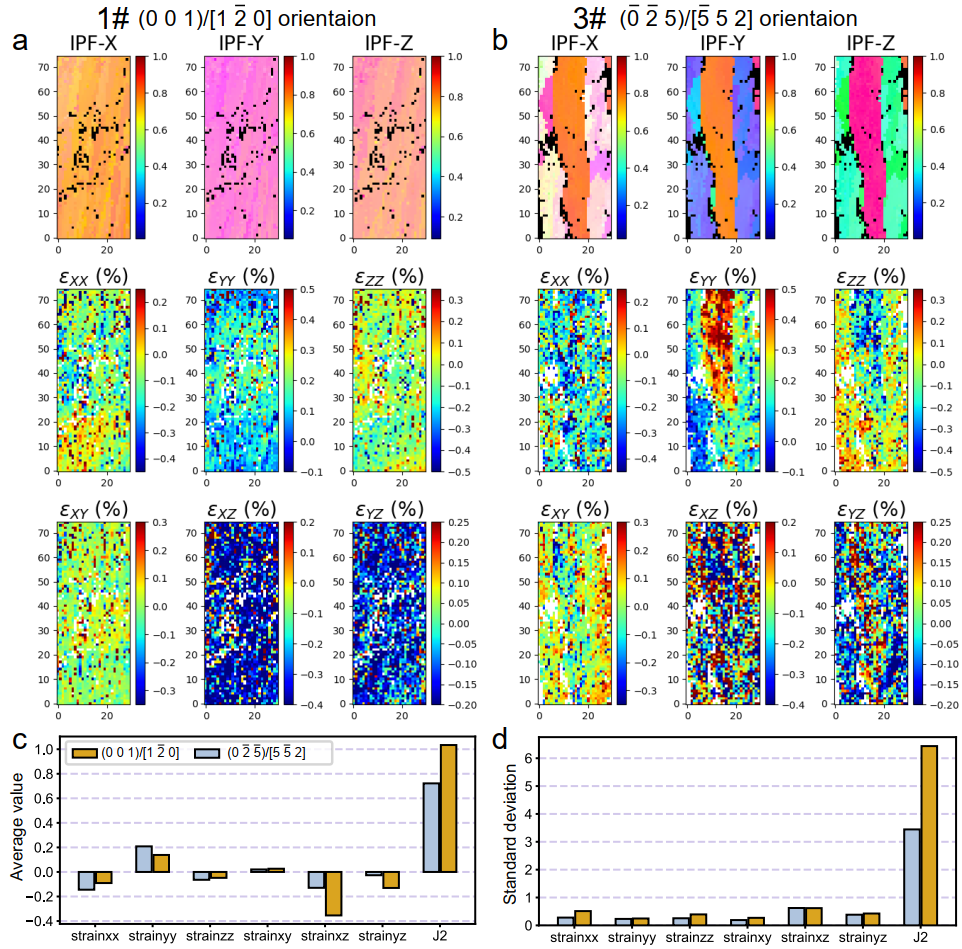


Figure S6.

**Spatial distribution map of orientation and deviatoric strain at the top of the printed region based on the results of μLaue diffraction experiments.** (**A**) 1#-(001)/[$1\bar{2}0$]. (**B**) 3#-($0\bar{2}\bar{5}$])/[$5\bar{5}2$]. The black pixels in the IPF represent the data that is not indexed.


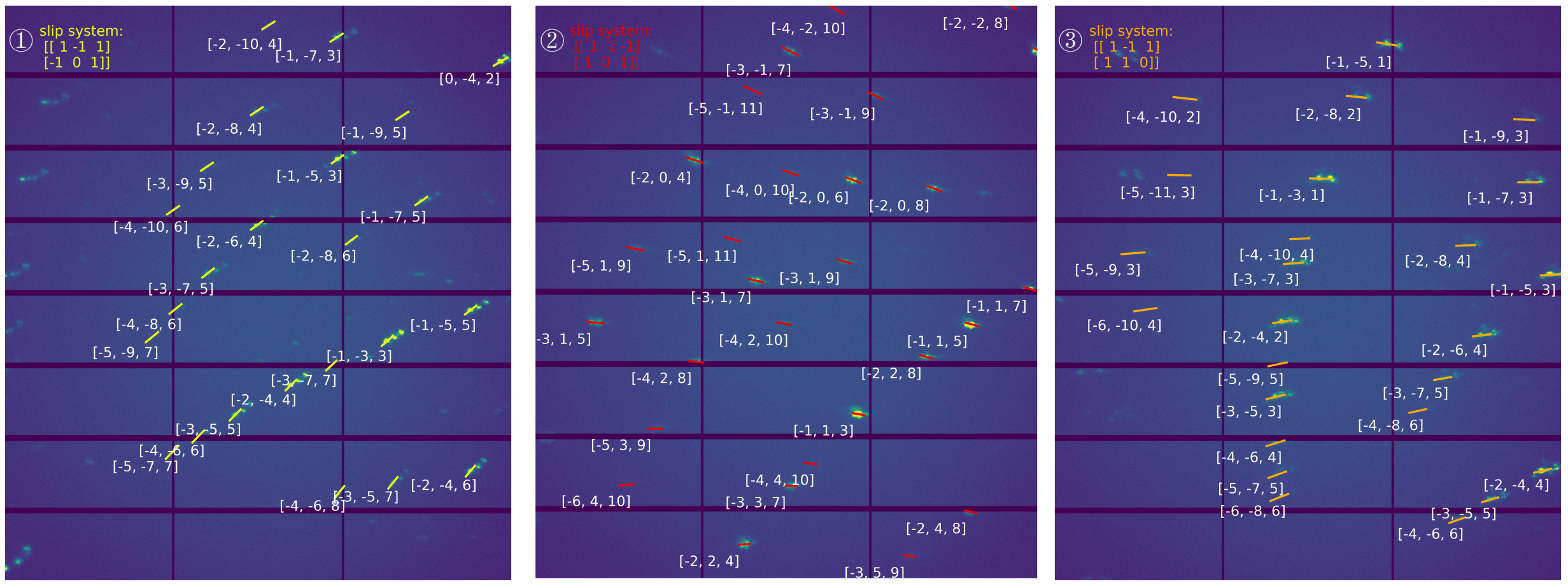


Figure S7.

**Simulated Laue diffraction patterns with activated dislocation slip systems of ①, ②, and ③ SGs shown in Figure 2H**.


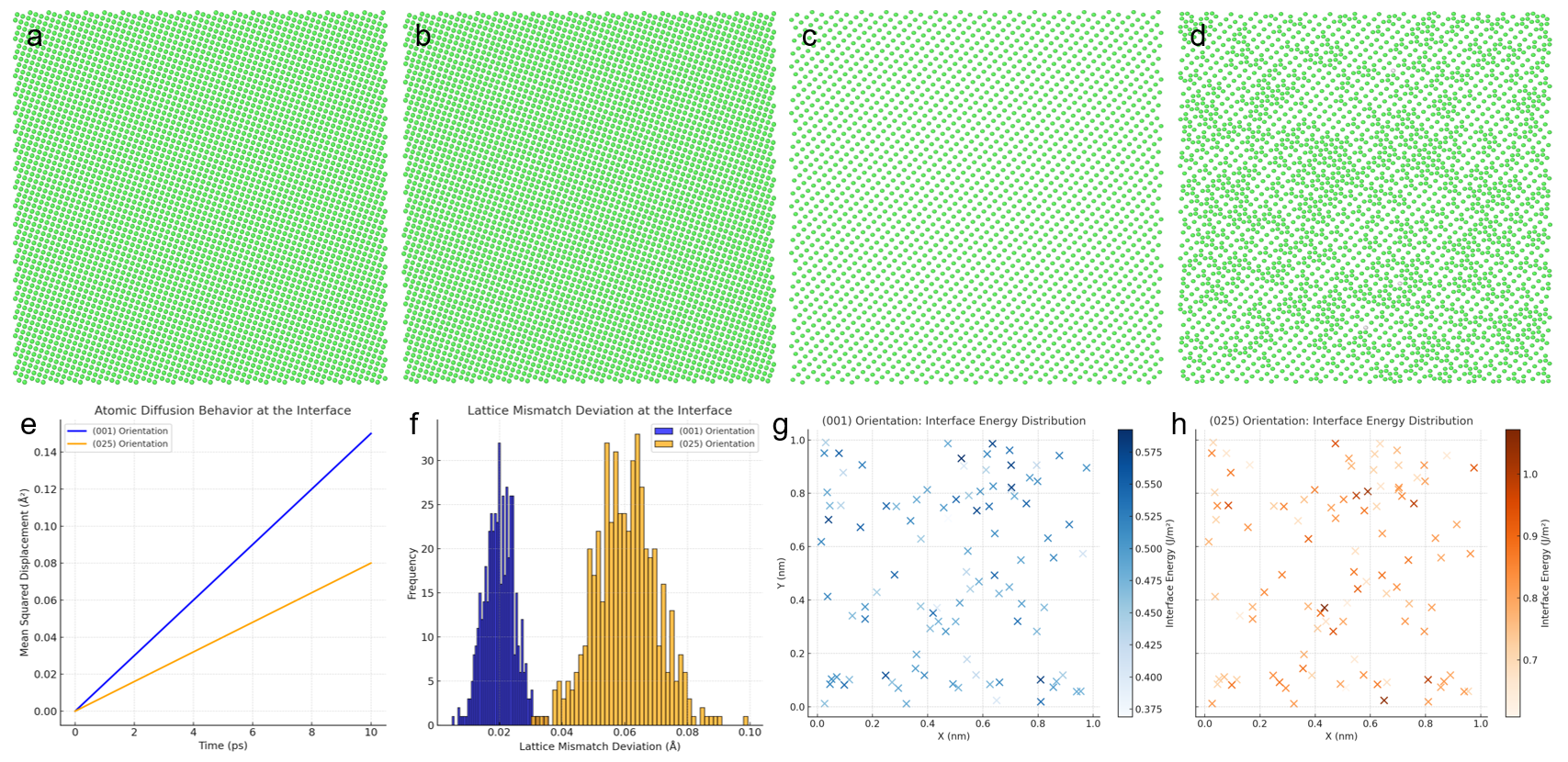


Figure S8.

**Analysis of atomic evolution behavior during the solidification process.** (**A-B**) Epitaxial crystal plane with initial (A) and relaxed (B) atomic arrangement of 1#-(001)/[$1\bar{2}0$] orientation. (**C-D**) Epitaxial crystal plane with initial (C) and relaxed (D) atomic arrangement of 3#-($0\bar{2}\bar{5}$])/[$5\bar{5}2$] orientation. (**E**) The variation of mean square displacement (MSD) of interface atoms over time. (**F**) Lattice matching statistics. (**G-H**) Epitaxial crystal plane energy statistics of 1#-(001)/[$1\bar{2}0$] (G)and #-($0\bar{2}\bar{5}$])/[$5\bar{5}2$] orientations (H).


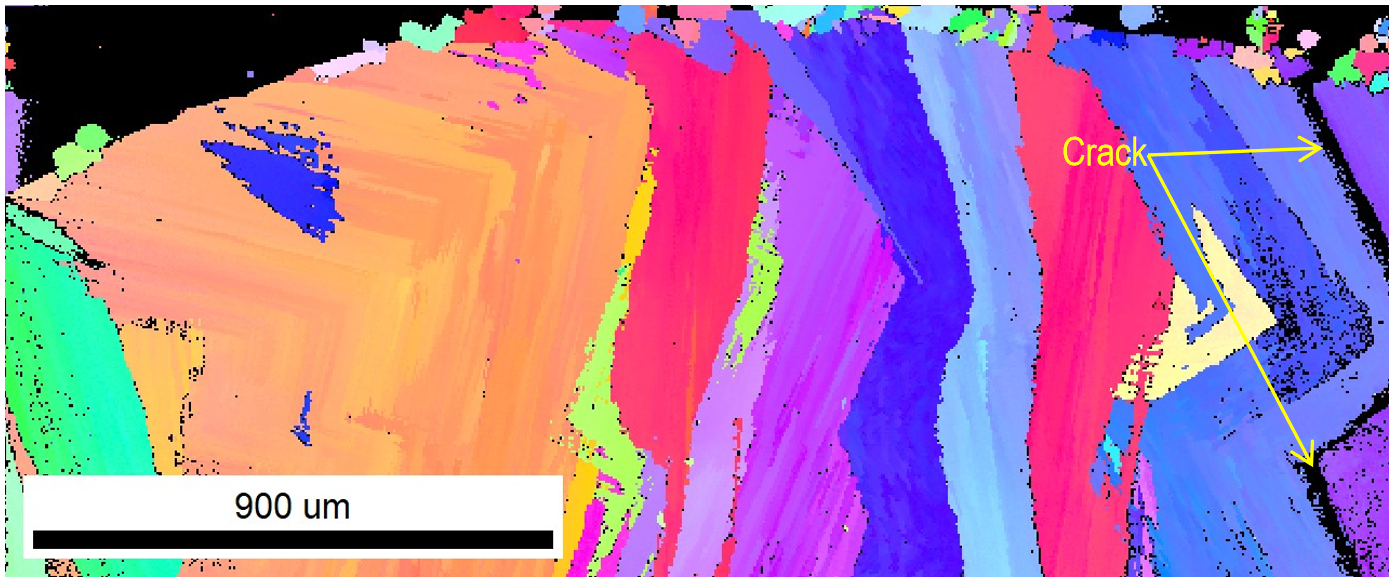


Figure S9.

**EBSD results of L-DEDed samples of 4#-($\mathbf{0}\bar{\mathbf{2}}\mathbf{1}$)/[$\bar{\mathbf{1}}\mathbf{00}$] substrate orientation (IPF-Z)**.


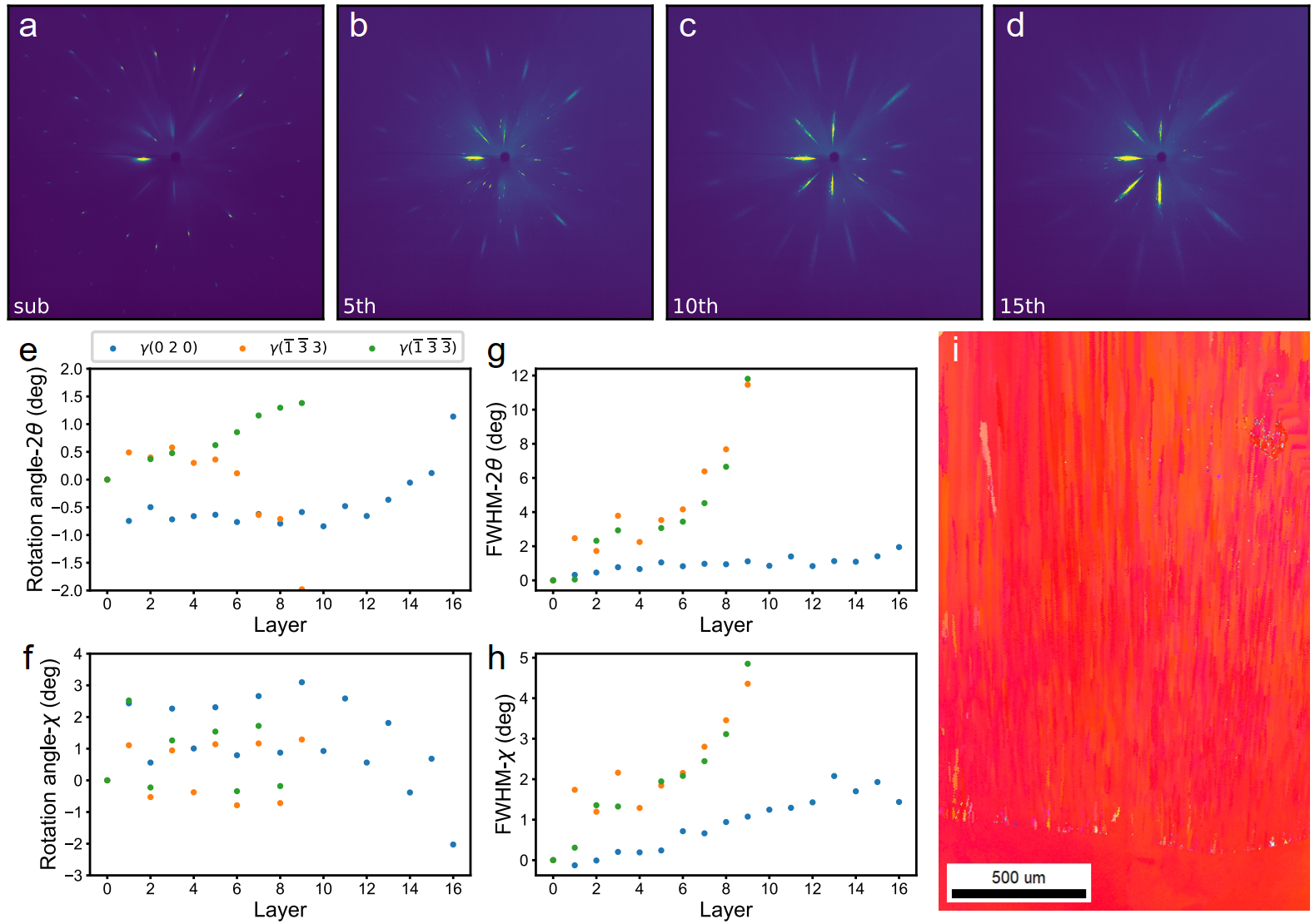


Figure S10.

**In situ Laue diffraction and EBSD results of (001)/[100] orientation**. (**A-D**) Laue patterns of substrate (A), the fifth layer (B), the tenth layer (C), and the fifteenth layer (D). (**E-F**) The rotation angle in the 2θ (E) and χ (F) direction versus time. (**G-H**) The FWHM in the 2θ (G) and χ (G) direction versus time. i, Orientation maps.


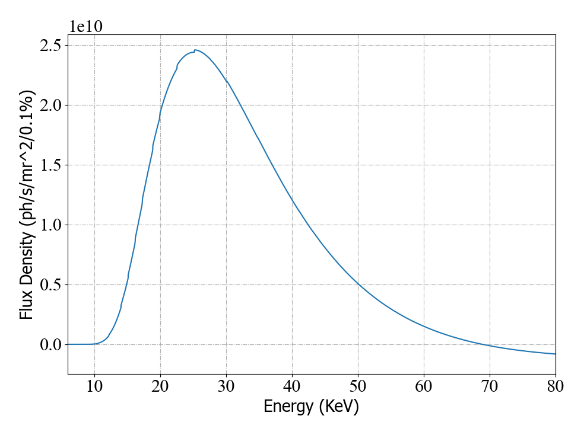


Figure S11.

**Energy spectrum of white beam X-ray generated from a superconducting wiggler.**


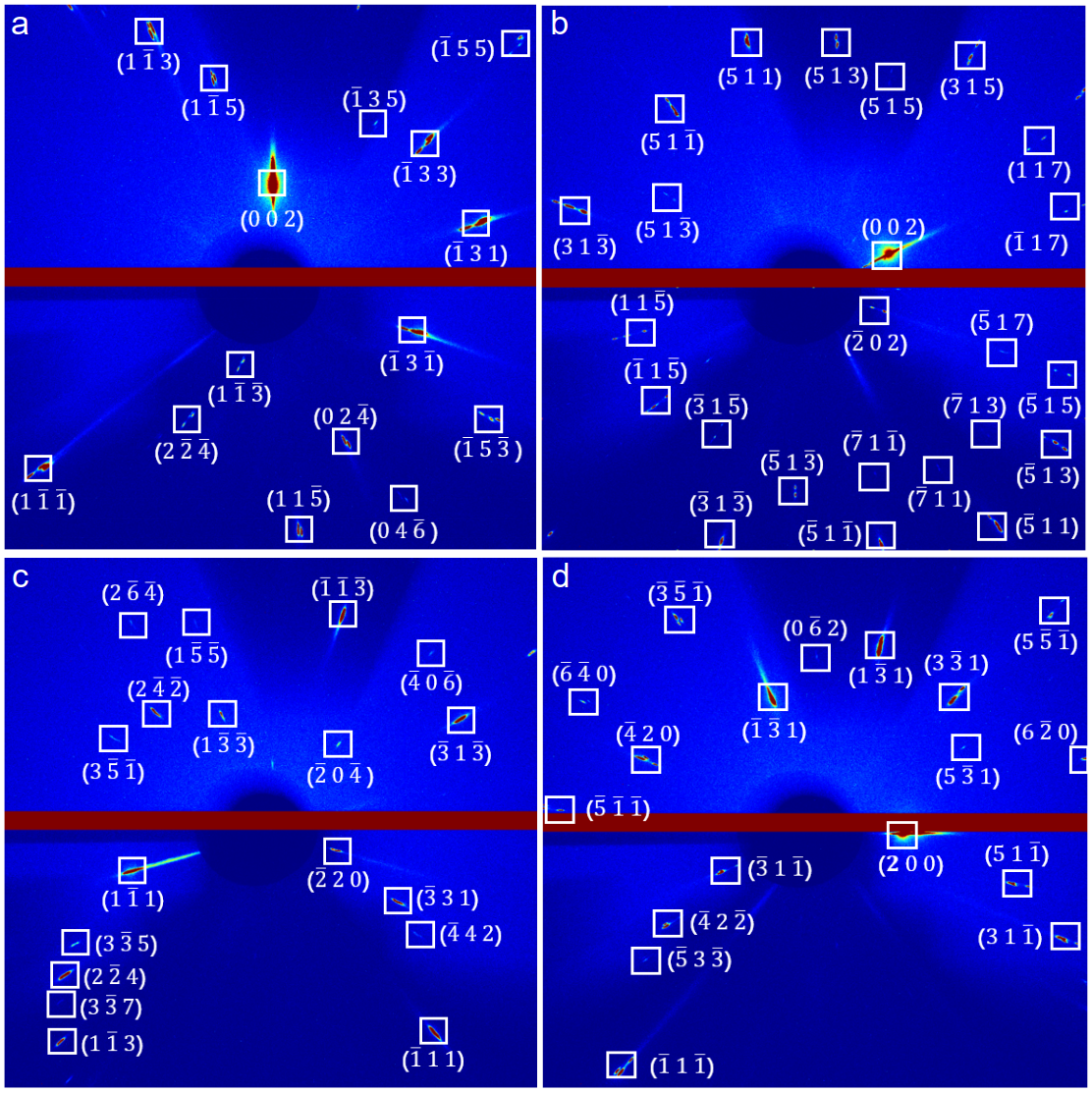


Figure S12.

**Laue diffraction patterns of four-orientation substrates**. (**A**) 1#-($001$)/[$1\bar{2}0$], (**B**) 2#-($201$)/[$10\bar{2}$], (**C**) 3#-($0\bar{2}\bar{5}$)/[$5\bar{5}2$] and (**D**) 4#-($0\bar{2}1$)/[$\bar{1}00$]. The Laue reflections are indexed in white.

Table S1.

**Orientation of substrates in the experimental coordinate system.**

| Sample | Orientation | X | Y | Z |
| --- | --- | --- | --- | --- |
| 1# | ($001$)/[$1\bar{2}0$] | ($1\bar{2}0$) | ($210$) | ($001$) |
| 2# | ($201$)/[$10\bar{2}$] | ($10\bar{2}$) | ($01 0$) | ($201$) |
| 3# | ($0\bar{2}\bar{5}$)/[$5\bar{5}2$] | ($5\bar{5}2$) | ($\bar{5}\bar{5}2$) | ($0\bar{2}\bar{5}$) |
| 4# | ($0\bar{2}1$)/[$\bar{1}00$] | ($\bar{1}00$) | ($0\bar{1}\bar{2}$) | ($0\bar{2}1$) |


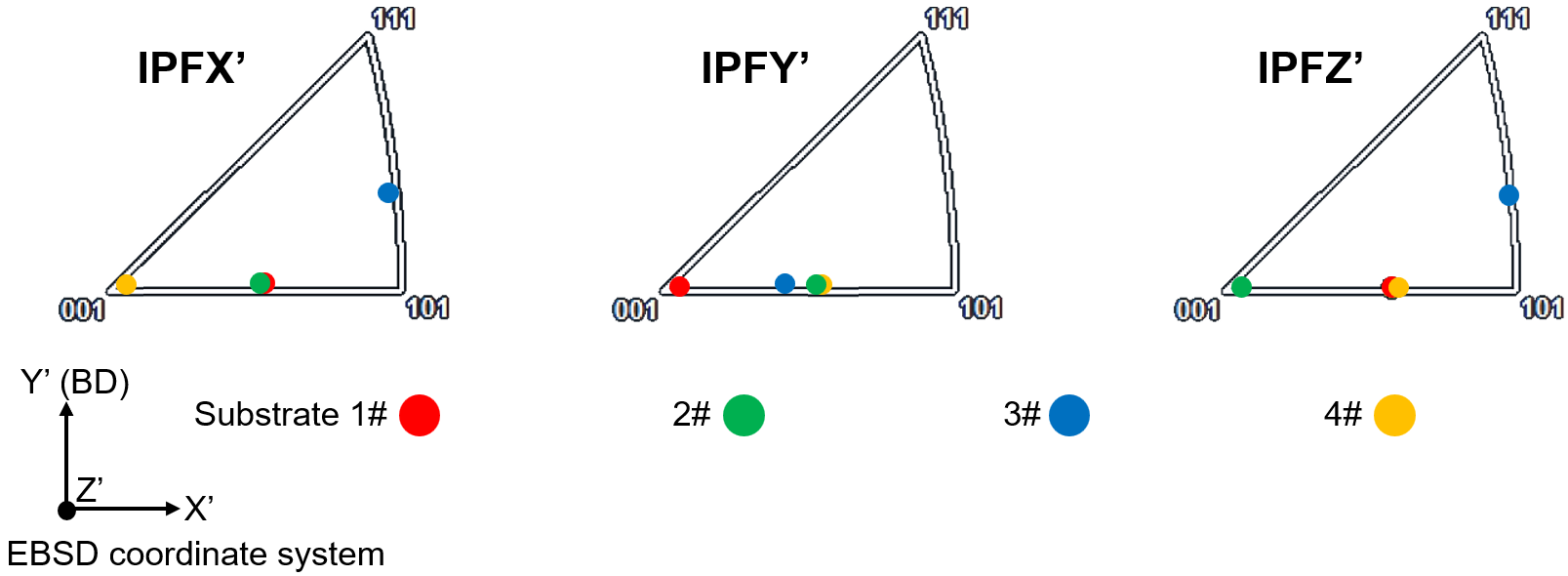


Figure S13.

**Inverse pole figures for the four orientations of substrates used in L-DED printing.**

Table S2.

**MD-calculated boundary energy and Gini index of different substrates.**

| Orientation | Boundary energy | Gini index |
| --- | --- | --- |
| ($\mathbf{001}$)/[$\mathbf{1}\bar{\mathbf{2}}\mathbf{0}$] | 0.495 J/m² | 0.169 |
| ($\mathbf{201}$)/[$\mathbf{10}\bar{\mathbf{2}}$] | 0.648 J/m² | 0.245 |
| ($\mathbf{0}\bar{\mathbf{2}}\bar{\mathbf{5}}$)/[$\mathbf{5}\bar{\mathbf{5}}\mathbf{2}$] | 0.802 J/m² | 0.380 |
| ($\mathbf{001}$)/[$\mathbf{100}$] | 0.485 J/m² | 0.142 |

Table S3.

**Chemical composition of the nickel-based single crystal superalloy (wt.%)**

| Co | Cr | Mo | W | Al | Ta | Hf | Re | Ni |
| --- | --- | --- | --- | --- | --- | --- | --- | --- |
| 7.5 | 7.0 | 1.5 | 5.0 | 6.2 | 6.5 | 0.15 | 3.0 | Bal. |

Table S4.

**The material properties of SX superalloy for the molten pool dynamic simulation**

| Material properties | Values |
| --- | --- |
| Density ($\rho$) | 8676 kg/m^3^ |
| Solidus temperature ($T_{s}$) | 1603 K |
| Liquidus temperature ($T_{l}$) | 1645 K |
| Boiling temperature ($T_{b}$) | 3017 K |
| Latent heat of melting ($h_{m}$) | J/kg |
| Latent heat of evaporation ($h_{e}$) | J/kg |
| Surface tension coefficient ($\sigma_{0}$) | 1.877 N/m |
| Temperature sensitivity of surface tension ($\sigma_{s}^{T}$) | N/(m K) |
| Viscosity ($\nu$) | $0.0085-1.02\times{10}^{-5}(T-1603)$ Pa s |

Table S5.

**The temperature-dependent specific heat and thermal conductivity of SX superalloy**

| Temperature (K) | Specific heat (J/kg K) | Thermal conductivity (W/m K) |
| --- | --- | --- |
| 300 | 411 | 11.42 |
| 500 | 445 | 13.69 |
| 700 | 476 | 15.85 |
| 900 | 573 | 17.98 |
| 1100 | 623 | 20.28 |
| 1300 | 745 | 23.59 |
| 1500 | 949 | 30.87 |
| 1603 | 612 | 34.73 |
| 1645 | 651 | 32.17 |
| 1800 | 677 | 34.80 |
| 2000 | 683 | 38.22 |

Table S6.

**The calibrated parameters used in the crystal-plasticity model**

|  | **Symbol** | **Value** | **Unit** | **Calibration method** |
| --- | --- | --- | --- | --- |
| Elastic properties | *C*_11_  *C*_12_  *C*_44_ | Refer to Figure S13B-C | GPa | The *C*_11_ and *C*_44_ in the crystal coordinate system are fitted using exponential functions, based on the experimental data from Ref. [1]. The Poisson’s ratio=0.39 is temperature-independent. |
| Thermal expansion | $\alpha$ | Refer to Figure S13D | 10^-6^ / K | Fitted using linear function, based on the experimental data from Ref. [1]. |
| Flow rule | *n* | 10 | - | Based on our previous work on Ni-based superalloys [2]. They are validated from room to elevated temperatures, using the experimental stress-strain curves. |
|  |  | 0.001 | s^-1^ |  |
|  | $\tau_{0}^{s}$ | Refer to Figure S13A | MPa | Fitted using an exponential function, based on the experimental yield strength from Ref. [1]. |
| Dislocation hardening |  | 0.15 | - | Based on our previous work on AM Ni-based single-crystal superalloys [3]. |
|  | *b* | 0.255 | nm | Based on our previous work on Ni-based superalloys [4]. |
|  | *y*_c_ | 0.016 | μm | Based on our previous work on Ni-based superalloys [2]. |
|  | *K* | 0.35 | - | Based on our previous work on AM Ni-based single-crystal superalloys [3]. |
|  | $\chi_{s}^{\alpha}$ | 1 (*α* = *β*), 1.4 (*α* ≠ *β*) | - | Based on our previous work on Ni-based superalloys [2]. |


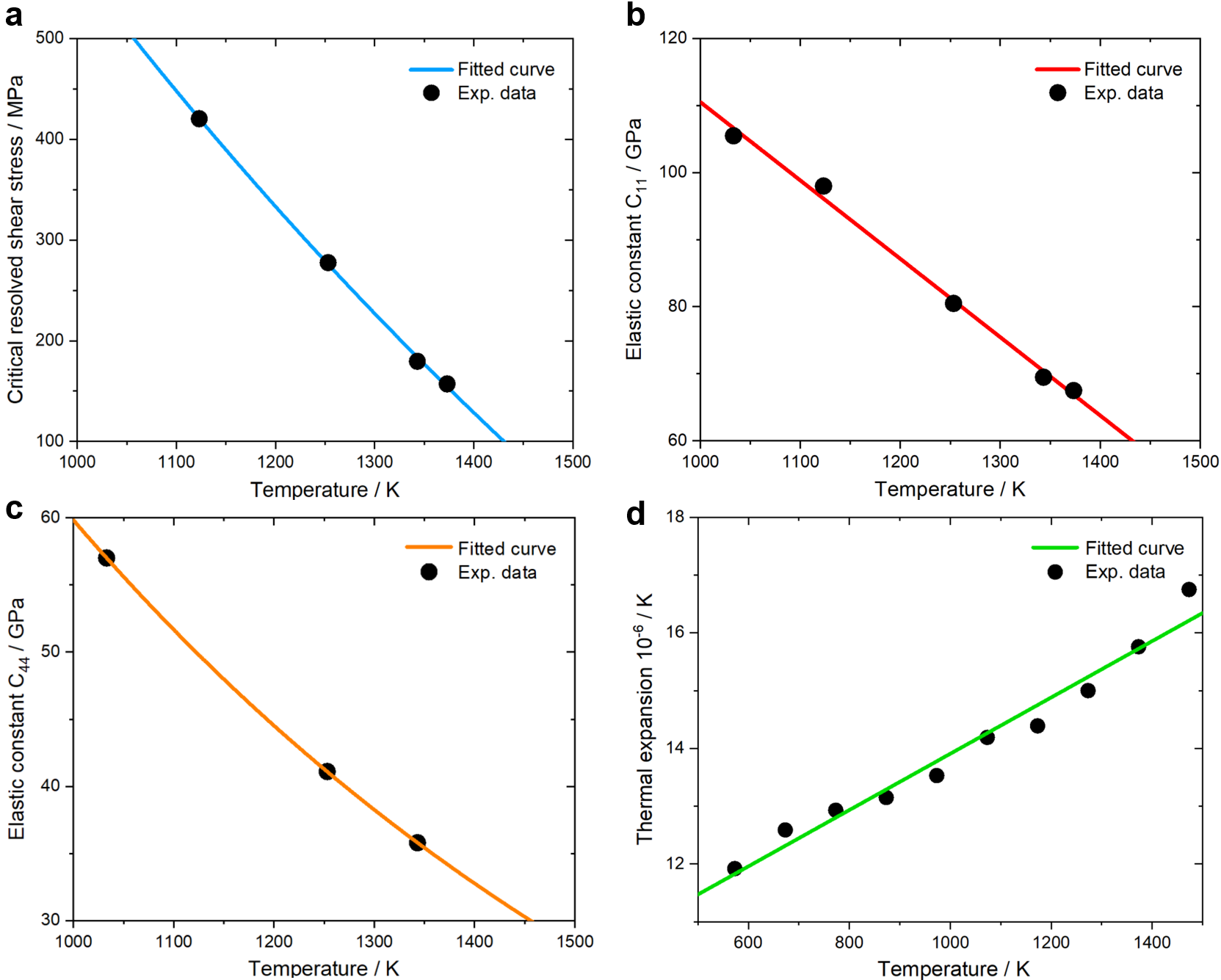


Figure S14.

**Temperature-dependent parameters in crystal-plasticity simulation.** **(A-C)** Critical resolved shear stress, elastic constants *C*_11_ and *C*_44_ (in crystal coordinate system), which are fitted using the exponential function y=a*exp(-x/b)+c. **(D)** Thermal expansion, which is fitted using a linear relationship. The experimental data are all taken from the property report of Ni-based single-crystal superalloy [1].


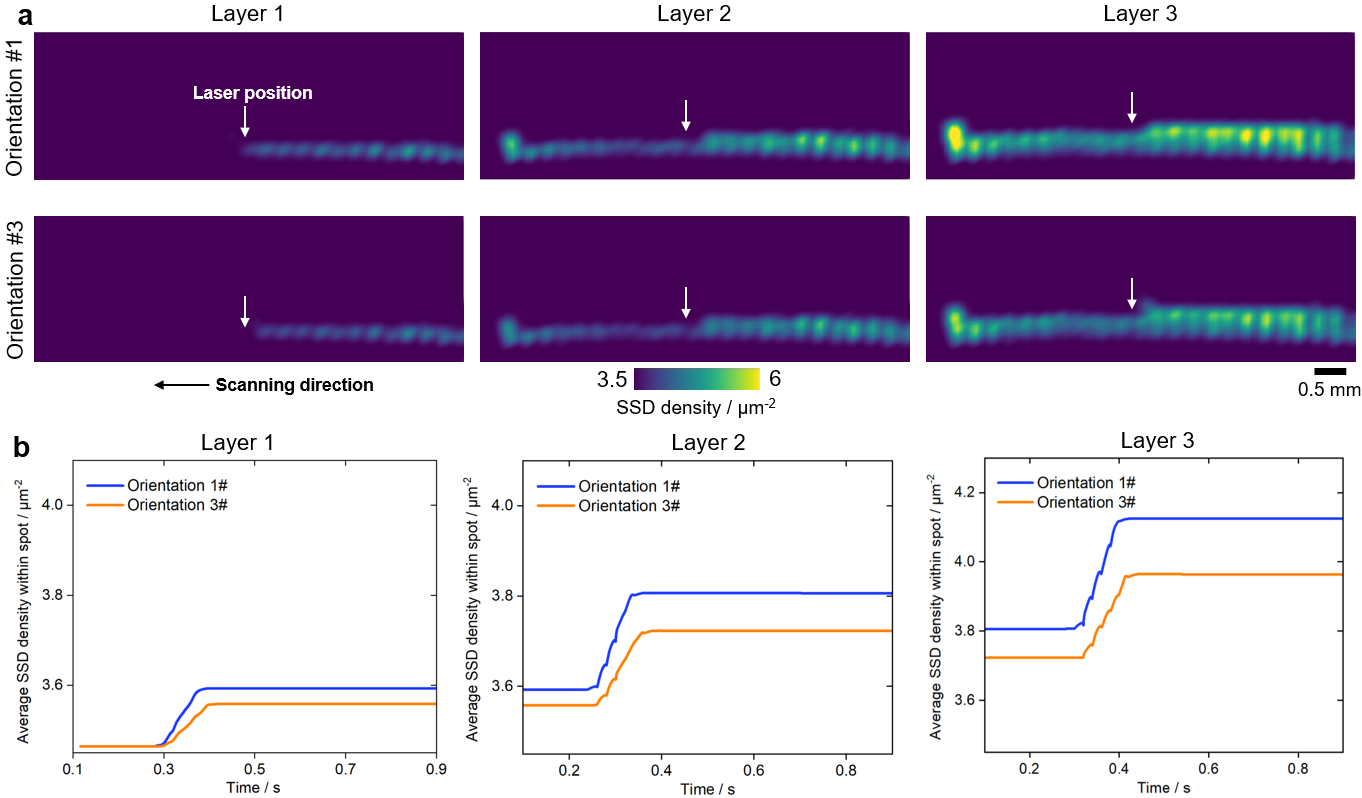


Figure S15.

**Distribution of SSD density in the first three layers during L-DED printing calculated using multi-physics modeling. (A)** Comparison of SSD patterns between orientations #1 and #3, showing that orientation #1 exhibits a higher SSD density throughout the L-DED process. **(B)** Evolution of the average SSD density within the beam spot, indicating that the difference in SSD density between orientations #1 and #3 becomes increasingly pronounced at the later stage of L-DED printing.

Supplementary Text 2

Diffraction peak width analysis

1. Energy bandwidth and instrument broadening:

As shown in Figure S11, the probe ‘white’ X-rays are generated from a superconducting wiggler, giving a high brilliance and a broad band spectrum. For synchrotron ‘white’ X-rays, the diffraction peak width caused by divergence is negligible. The broadening of detectors and other instruments remains constant in the experiment, thereby having minimal effect on the changes in peak width with time.

2. Grain size effect:

Based on the Scherrer formula, the smaller the grain size corresponds to greater the broadening and vice-versa. When the grain size reaches the micron-level, the peak width caused by the grain size effect can be ignored as it is controlled by other factors. For AM metals with columnar grains, as indicated by Figure S4, the size of solidified grain perpendicular to the build direction (X- and Y-axis) are in the micron-scale, while that along the build direction (Z-axis) is larger. Thus, the contribution of grain size to peak width can be ignored.

3. Strain gradients:

To quantify the contribution of strain gradient to peak width, the strain gradient calculated using a multi-physics model is inputted into the diffraction simulation, which indicates that the strain gradient has minimal contribution to the diffraction peak broadening. Calculation details are provided below.

Step 1: The diffraction simulation is used to obtain the crystal orientation from the Laue diffraction patterns and the FWHM of diffraction spots induced by dislocation and micro-strain.

Step 2: To exactly obtain the single crystal orientation relative to the incident X-ray, we use forward simulation of the Laue diffraction to index our X-ray diffraction patterns with the Miller indices [5, 6]. Given the known information including the experimental geometry and the “white beam” synchrotron X-ray source spectrum, we enumerate all possible orientations and calculate the corresponding X-ray diffraction patterns captured by the detector, which are compared with a measured diffraction pattern to find the best match [7, 8].

Step 3: First, considering the size of the X-ray spot of 100 μm in the experiment, a crystal calculation area of 100 × 100 × 100 μm^3^ is given, where the grid computing cells are divided into 25 × 25 × 25, and each of which is set to an ideal single crystal. Then, according to the strain gradient obtained from thermomechanical simulation, the strain tensor at different positions is calculated and applied to each computing cell to deform the single crystal, based on which we can obtain the simulated diffraction pattern under the complex strain case. The contribution of the dislocation density to the peak width is calculated based on the modified Williamson-Hall method [9-11].

Step 4: The simulation allowed us to track the evolution of FWHM induced by the strain gradient over time, as illustrated in Figure S16. The diffraction peak width remains within a very narrow range (< 0.04°), significantly smaller than the experimental results in Figure 2. Therefore, the strain gradient has a negligible contribution to the observed diffraction broadening.


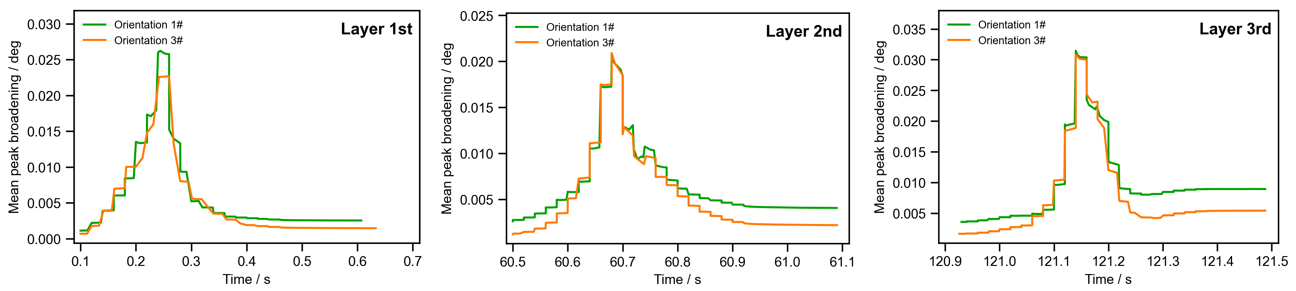


Figure S16.

**Evolution of peak broadening contributed by the strain gradient during solidification.** The green and orange curves represent orientations 1# and 3#, respectively.

Supplementary Text 3

Laue spot number analysis

Since the orientations of SGs differ from those of epitaxially grown grains, a higher SG content leads to an increased number of discrete Laue diffraction spots. To pick out the number of diffraction spots, the intensity threshold and area threshold of diffraction spots are set to 60 and 4 pixels, respectively. The area threshold is utilized to avoid the errors caused by the dead pixel and single-pixel statistical fluctuation of counts. The intensity threshold is used to avoid the influence of unmelted powder, as the diffraction intensity of the powder is low. The experimental results of other orientations (Figure S1) and reference [12] also indicate that this method is feasible for qualitative characterization of impurity content.

To further demonstrate the effectiveness of using the number of diffraction spots for the qualitative characterization of SG content in synchrotron diffraction analysis, we have conducted additional analysis on the ex-situ EBSD results. As shown in Figure S17A, there are fewer HAGBs in L-DED printing under substrate orientation 1#, corresponding to a minimal increased number of diffraction peaks in synchrotron diffraction (Figure 2A). In contrast, during the multi-layer printing on orientation 3# (Figure S17B), HAGB suddenly comes into being after 10 layers, which matches well with the rapid increase in the number of diffraction peaks shown in Figure 2B. Therefore, EBSD analysis of the multi-layer printed part confirms that the number of diffraction spots serves as a rational indicator for the formation of SGs in synchrotron diffraction analysis.


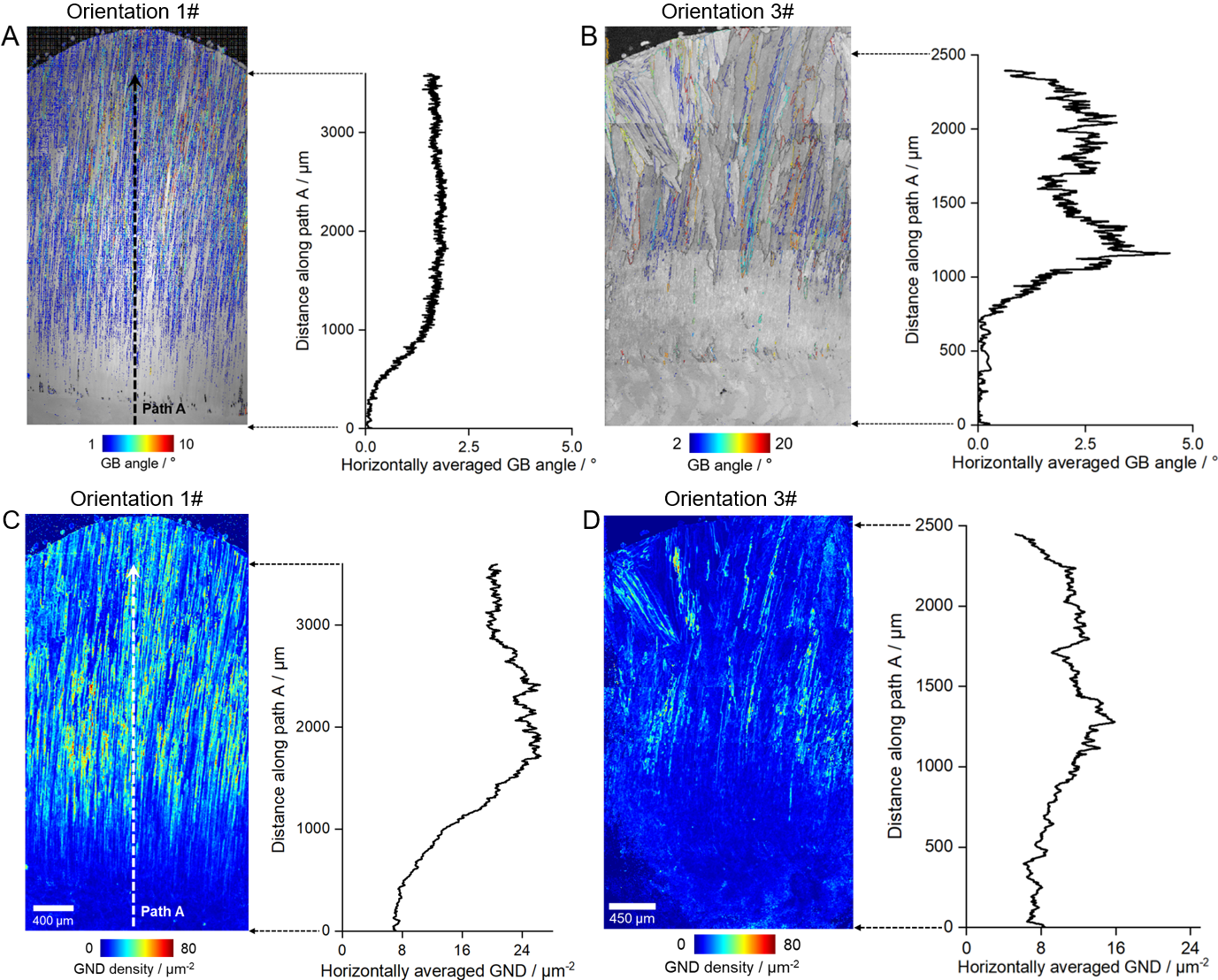


Figure S17.

**EBSD-derived HAGB and GND density distributions.** Results for orientation 1# **(A, C)** and orientation 3# **(B, D)**, where the GB angles and GND density are horizontally averaged in the plotted curves, to represent the overall relationship between GB angles/GND density and building height.

Supplementary Text 4

Gini coefficient calculation

(1) Data source (MD-based)

In this study, the Gini coefficient is computed directly from molecular dynamics (MD) simulation outputs. Specifically, the input data is the local dislocation density obtained in post-processing using the Dislocation Extraction Algorithm (DXA). For each spatial bin, we first accumulate the total dislocation length contained in that bin and then normalize it by the bin volume, yielding a voxel-scale dislocation density field ρ(x) (unit: m^-2^), where x denotes the spatial coordinates. The Gini coefficient is subsequently calculated from the distribution of ρ over all bins within the defined ROI.

(2) Spatial binning strategy (voxelization)

To ensure consistency and comparability among different crystallographic orientations, the simulation domain is discretized into a regular three-dimensional voxel grid using a uniform bin size for all cases. For each voxel v, the scalar used for the Gini calculation is

ρ_v_ = L_v_ / V_v_

where L_v_ is the total DXA-extracted dislocation line length within voxel v, and V_v_ is the voxel volume. In our post-processing, a voxel size of approximately 5 nm × 5 nm × 5 nm is employed (the overall MD domain size for each orientation is hundreds of nanometres). Importantly, the same voxelization settings are applied to all cases, ensuring that the variation in Gini coefficients solely reflects changes in dislocation heterogeneity at different substrate orientations, rather than changes in binning.

(3) Region of interest (ROI)

The Gini coefficient is evaluated in the solidified region after solidification, with emphasis on the interface/epitaxial growth zone where dislocations accumulate and subgrain boundaries form. We further define the ROI based on local structural state (solid vs. liquid) and position relative to the liquid-solid interface. Concretely, the ROI is identified using Common Neighbor Analysis (CNA): voxels in which the crystalline fraction exceeds 90% are treated as predominantly solid. The Gini statistics are then computed for the region of 20 voxel thickness behind this solidification front (i.e., immediately in the solidified track). This selection ensures that the reported Gini captures the heterogeneity of dislocation storage in the solidified/epitaxial region, rather than transient fluctuations in the liquid.

(4) Statistical time (snapshot/solidification stage)

The Gini coefficient is reported at a defined solidification stage, after the epitaxial layer has formed and the dislocation structure is largely stabilized. For the present solidification/cooling process (total duration 500 ps), we compute the metric using the snapshot at 400 ps, at which the dislocation structure is essentially stable (as shown in Figures 4C and 4F). The same timestep criterion is applied to all simulation cases, ensuring a consistent comparison.

Supplementary Text 5

EBSD-based GND calculation algorithm

To determine the Nye tensor $\alpha$ using EBSD data, $\alpha$ can be expressed by the local lattice curvature tensor $\kappa$ under small deformation conditions [13]:

where is the misorientation angle regarding *i* axis, $\varepsilon_{ij}^{\text{el}}$ is the elastic strain, $\upsilon_{klj}$ is the Levi–Civita permutation symbol. When calculating the spatial gradient of in the above equation, we employ the region of 3*3 pixels to compute the local gradient on the individual pixels. Moreover, in the above Nye tensor equation, the elastic strain gradients are negligible [14]. In the two-dimensional EBSD, there are 5 accessible components in the Nye tensor when using EBSD data as an input [13]: $\alpha_{12}=\kappa_{21}$, $\alpha_{13}=\kappa_{31}$, $\alpha_{21}=\kappa_{12}$, $\alpha_{23}=\kappa_{32}$, $\alpha_{33}=-\kappa_{11}-\kappa_{22}$. Finally, the 2nd norm of Nye tensor is used to represent the GND density:

$$\rho_{\text{GND}}=\frac{\sqrt{\sum_{j=1}^{3} \sum_{i=1}^{3} \alpha_{ij}^{2}}}{b}$$

where *b* represents the norm of the Burgers vector for the dislocation in {111}<110> slip systems.

Supplementary Text 6

Comparison with the constitutional supercooling mechanism

The classic constitutional supercooling mechanism (thermally CET-based interpretations) demonstrates the formation of equiaxed SGs subjected to the homogeneous and random nucleation on the top of the melt pool (Figure S18A), which is attributed to the lower temperature gradient and larger supercooling region ahead of the solidification front, compared with the bottom of the melt pool [15, 16].


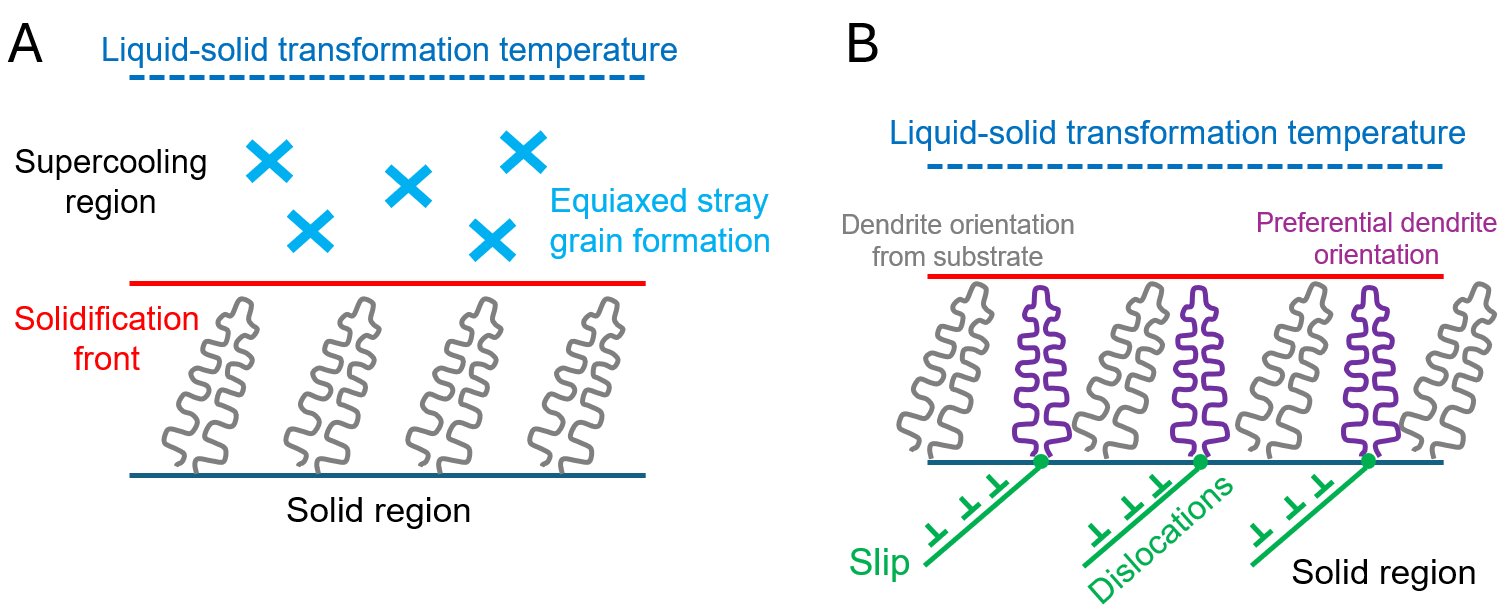


Figure S18.

**Schematic diagram for the mechanisms of SG formation in classic supercooling theory and our proposed deformation-sensitive mechanism.** **(A)** The equiaxed SGs tend to randomly nucleate within the larger supercooling region at the top of the melt pool with a lower temperature gradient. **(B)** Dislocation-induced columnar SG nucleation at the bottom of the melt pool with a higher temperature gradient and a smaller supercooling region, where the newly nucleated [001] dendrites with the highest growth rate are preferential during the solidification, which is consistent with the observation in Figure 3E.

In our proposed dislocation-sensitive mechanism, firstly, the temperature distribution of the melt pool should be unchanged across different substrate orientations using identical printing parameters. Hence, according to the classic constitutional supercooling mechanism, the random equiaxed SG formation at the supercooling regions should be independent with substrate orientations. However, our experiments show a substrate orientation-dependent columnar SG formation behavior, where the classic supercooling mechanism is not applicable. Secondly, in multi-layer printing under identical printing parameters and temperature distribution layer by layer (the parts are fully cooled down after the completion of each layer), the constitutional supercooling mechanism alone is not capable of understanding the in-situ observation result for orientation 3#, where columnar SGs are absent in the initial 10 layers but suddenly appear in subsequent ones (Figure S17B and D). In response, we adopt the dislocation density as a layer-wise cumulative quantity to serve as an indicator of columnar SG nucleation at the bottom of the melt pool (Figure S18B). Then, we demonstrate that the Gini coefficients of interface dislocations provide better prediction than the classic supercooling mechanism (Table S2 indicating the highest Gini coefficient for 3#). These two pieces of experimental evidence demonstrate that the nucleation of columnar SGs in this paper is not fully attributed to the classic constitutional mechanism.

Movie S1.

Evolution of Laue diffraction patterns during L-DED process of SX nickle-based superalloy corresponding to 1#-($001$)/[$1\bar{2}0$] orientation at laser power of 238 W and scan speed of 800 mm/min.

Movie S2.

Evolution of Laue diffraction patterns during L-DED process of SX nickle-based superalloy corresponding to 2#-($201$)/[$10\bar{2}$] orientation at laser power of 238 W and scan speed of 800 mm/min.

Movie S3.

Evolution of Laue diffraction patterns during L-DED process of SX nickle-based superalloy corresponding to 3#-($0\bar{2}\bar{5}$)/[$5\bar{5}2$] orientation at laser power of 238 W and scan speed of 800 mm/min.

Movie S4.

Evolution of Laue diffraction patterns during L-DED process of SX nickle-based superalloy corresponding to 4#-($0\bar{2}1$)/[$\bar{1}00$] orientation at laser power of 238 W and scan speed of 800 mm/min.

Movie S5.

Temperature evolution during the first three layers L-DED process for 1#-($001$)/[$1\bar{2}0$] orientation calculated by the thermo-fluid coupling simulation.

Movie S6.

J2 evolution during the first three layers L-DED process for 1#-($001$)/[$1\bar{2}0$] orientation calculated by the thermo-fluid-crystal plasticity coupling simulation.

Movie S7.

GND evolution during the first three layers L-DED process for 1#-($001$)/[$1\bar{2}0$] orientation calculated by the thermo-fluid-crystal plasticity coupling simulation.

**Reference**

[1] X.W. Huichen Yu, Materials Data Manual in Aircraft Engine Design (4th Edition), Chinese Aviation Industy Press 2010.

[2] Z. Zhang, Z. Guo, Q. Han, D. Hu, S. Wu, H. Fan, E. Li, M. Li, Y. Xu, S. Yang, C. Huang, W. Yan, Anomalous anisotropy in an additively manufactured solid-solution-strengthened superalloy from room to elevated temperatures, International Journal of Plasticity 192 (2025) 104409.

[3] Z. Guo, Y. Li, L. Fan, S. Wu, D. Hu, G. Peng, F. Lin, Y.-W. Zhang, Y. Xu, W. Yan, Trigonometric gradient microstructures in additively manufactured single crystals enable strength-ductility synergy and programmable performance, Nature Communications 16(1) (2025) 9936.

[4] Z. Guo, Z. Song, H. Liu, D. Hu, D. Huang, X. Yan, W. Yan, A dislocation-based damage-coupled constitutive model for single crystal superalloy: Unveiling the effect of secondary orientation on creep life of circular hole, International Journal of Plasticity 173 (2024) 103874.

[5] X. Huang, LauePt, a graphical-user-interface program for simulating and analyzing white-beam X-ray diffraction Laue patterns, Applied Crystallography 43(4) (2010) 926-928.

[6] J. Huang, Y. Zhang, S. Hu, Y. Cai, S. Luo, DATAD: a Python-based X-ray diffraction simulation code for arbitrary texture and arbitrary deformation, Applied Crystallography 54(2) (2021) 686-696.

[7] Y. Zhang, Y. Li, D. Fan, N. Zhang, J. Huang, M. Tang, Y. Cai, X. Zeng, T. Sun, K. Fezzaa, Ultrafast x-ray diffraction visualization of b 1-b 2 phase transition in kcl under shock compression, Physical review letters 127(4) (2021) 045702.

[8] V.K. Gupta, S.R. Agnew, Indexation and misorientation analysis of low-quality Laue diffraction patterns, Applied Crystallography 42(1) (2009) 116-124.

[9] T. Ungár, A. Borbély, The effect of dislocation contrast on x‐ray line broadening: A new approach to line profile analysis, Applied Physics Letters 69(21) (1996) 3173-3175.

[10] T. Ungár, S. Ott, P. Sanders, A. Borbély, J. Weertman, Dislocations, grain size and planar faults in nanostructured copper determined by high resolution X-ray diffraction and a new procedure of peak profile analysis, Acta materialia 46(10) (1998) 3693-3699.

[11] T. Ungár, I. Dragomir, Á. Révész, A. Borbély, The contrast factors of dislocations in cubic crystals: the dislocation model of strain anisotropy in practice, Applied Crystallography 32(5) (1999) 992-1002.

[12] D. Zhang, W. Liu, Y. Li, D. Sun, Y. Wu, S. Luo, S. Chen, Y. Tao, B. Zhang, In situ observation of crystal rotation in Ni-based superalloy during additive manufacturing process, Nature Communications 14(1) (2023) 2961.

[13] W. Pantleon, Resolving the geometrically necessary dislocation content by conventional electron backscattering diffraction, Scripta Materialia 58(11) (2008) 994-997.

[14] Z. Guo, X. Lu, C. Paramatmuni, H. Gao, F.P.E. Dunne, W. Yan, Y.-W. Zhang, Y. Xu, Slip system-resolved GNDs and SEDs: A multi-scale framework for predicting crack nucleation in single-crystal metals, Acta Materialia 288 (2025) 120853.

[15] W. Kurz, D. Fisher, M. Rappaz, Fundamentals of solidification, (2023).

[16] J. Zhao, J. Zheng, H. Huang, W. Zhong, Formation of low angle boundaries and stray grains in welds of single-crystal Mo-3Nb alloys, Science and Technology of Welding and Joining 25(4) (2020) 320-328.
