# Supplementary figures and images for: Deformation‐Induced Formation of Stray Grains in Additive Manufacturing of Single Crystals
Source: Adv Sci (Weinh). 2026 Feb 15;13(25):e22704. doi: 10.1002/advs.202522704 (PMC13137851; doi:10.1002/advs.202522704)

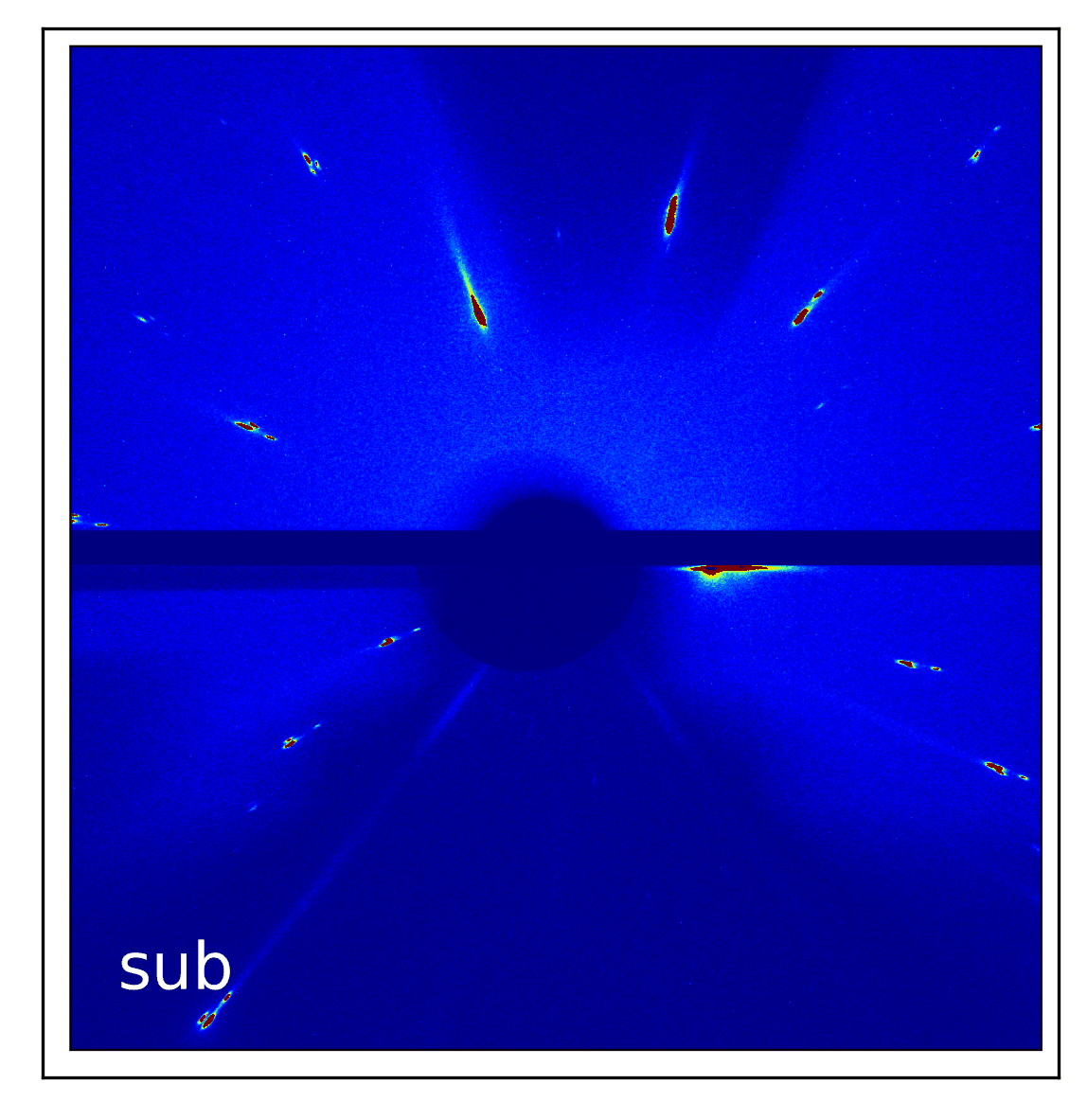

Supplement: Supplementary file 9 — Supporting file 9: advs74236‐sup‐0009‐Movies.zip. [file ADVS-13-e22704-s005.zip › Movies S4.gif]

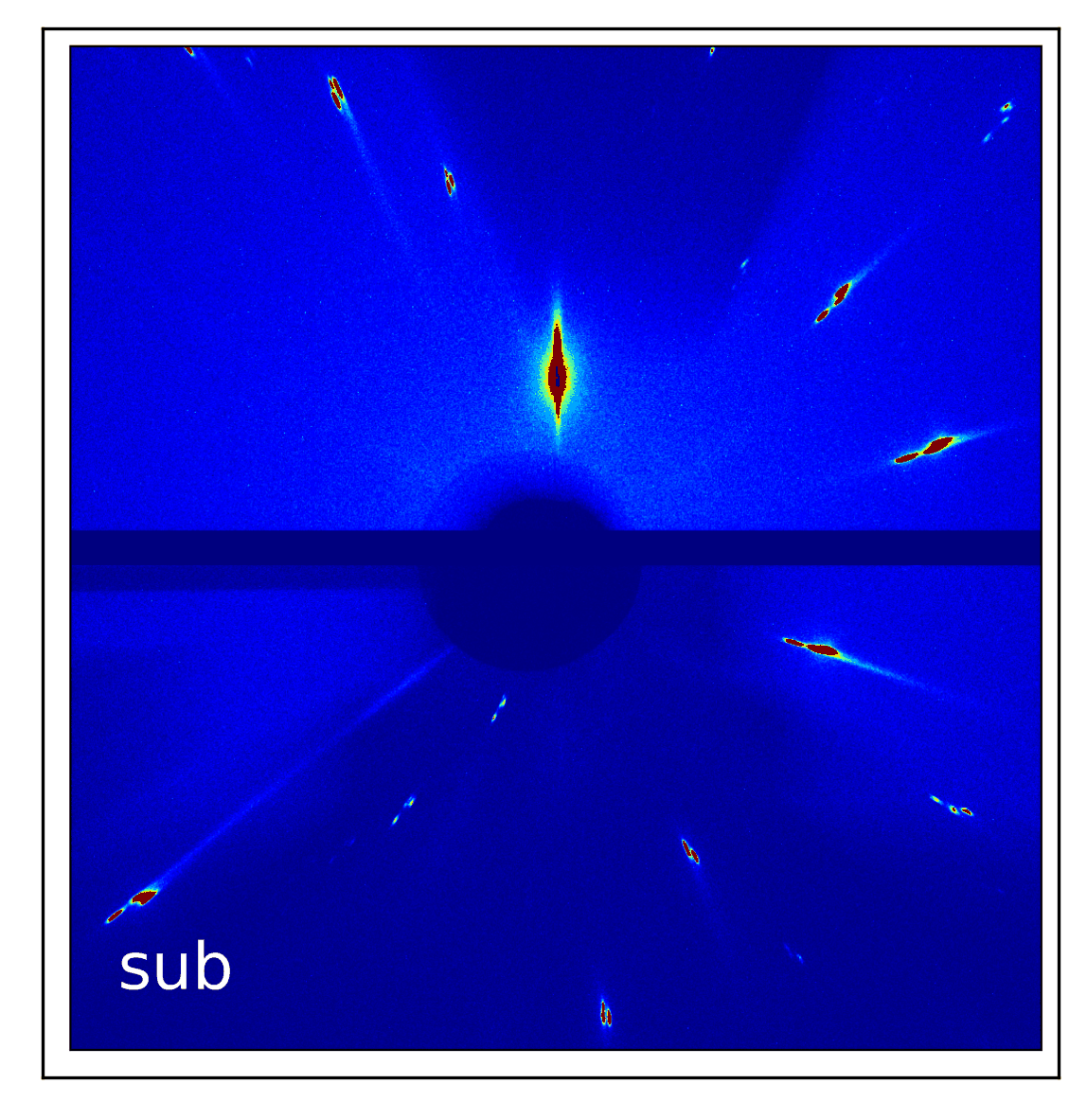

Supplement: Supplementary file 9 — Supporting file 9: advs74236‐sup‐0009‐Movies.zip. [file ADVS-13-e22704-s005.zip › Movies S1.gif]

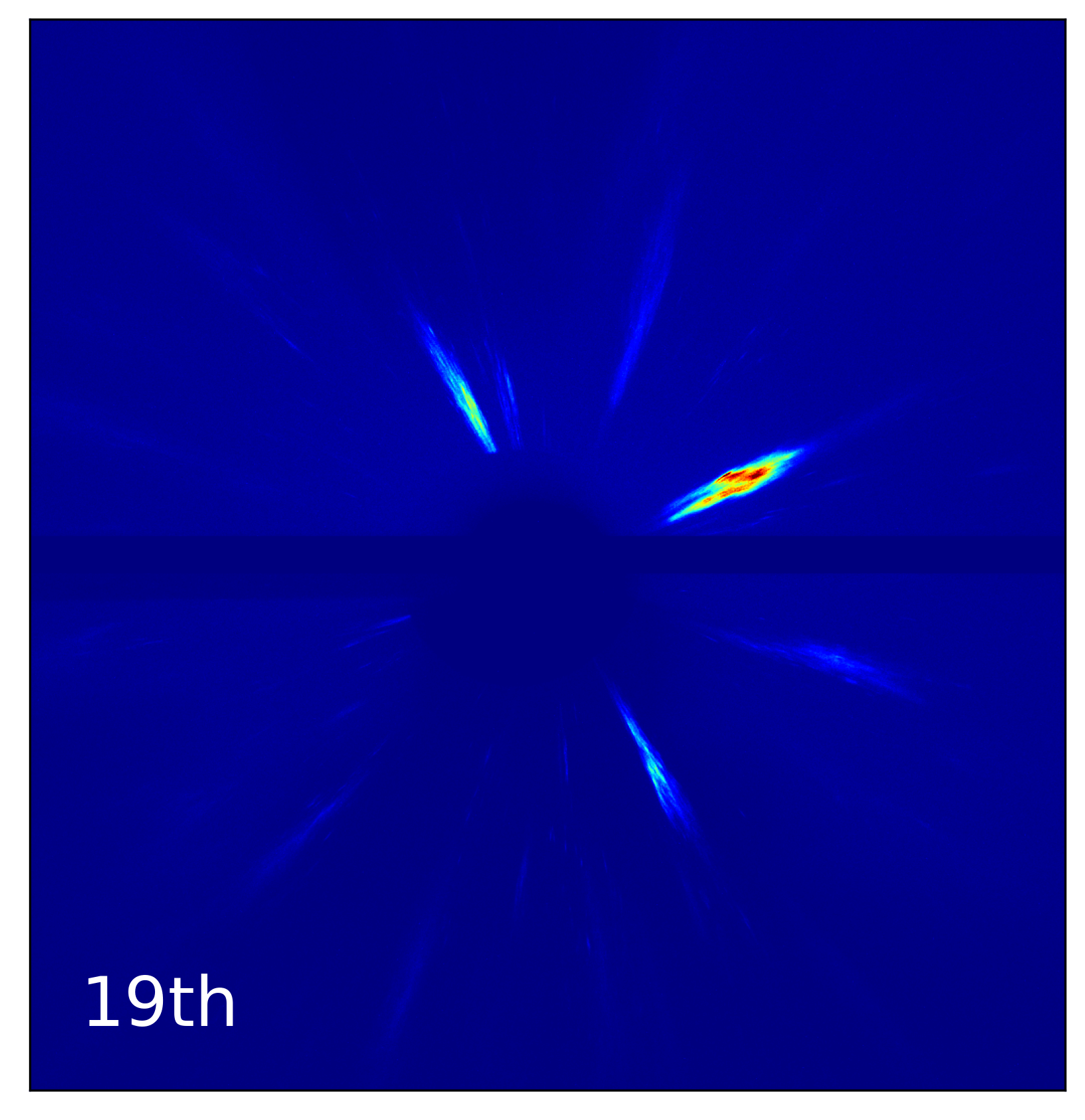

Supplement: Supplementary file 9 — Supporting file 9: advs74236‐sup‐0009‐Movies.zip. [file ADVS-13-e22704-s005.zip › Movies S2.gif]

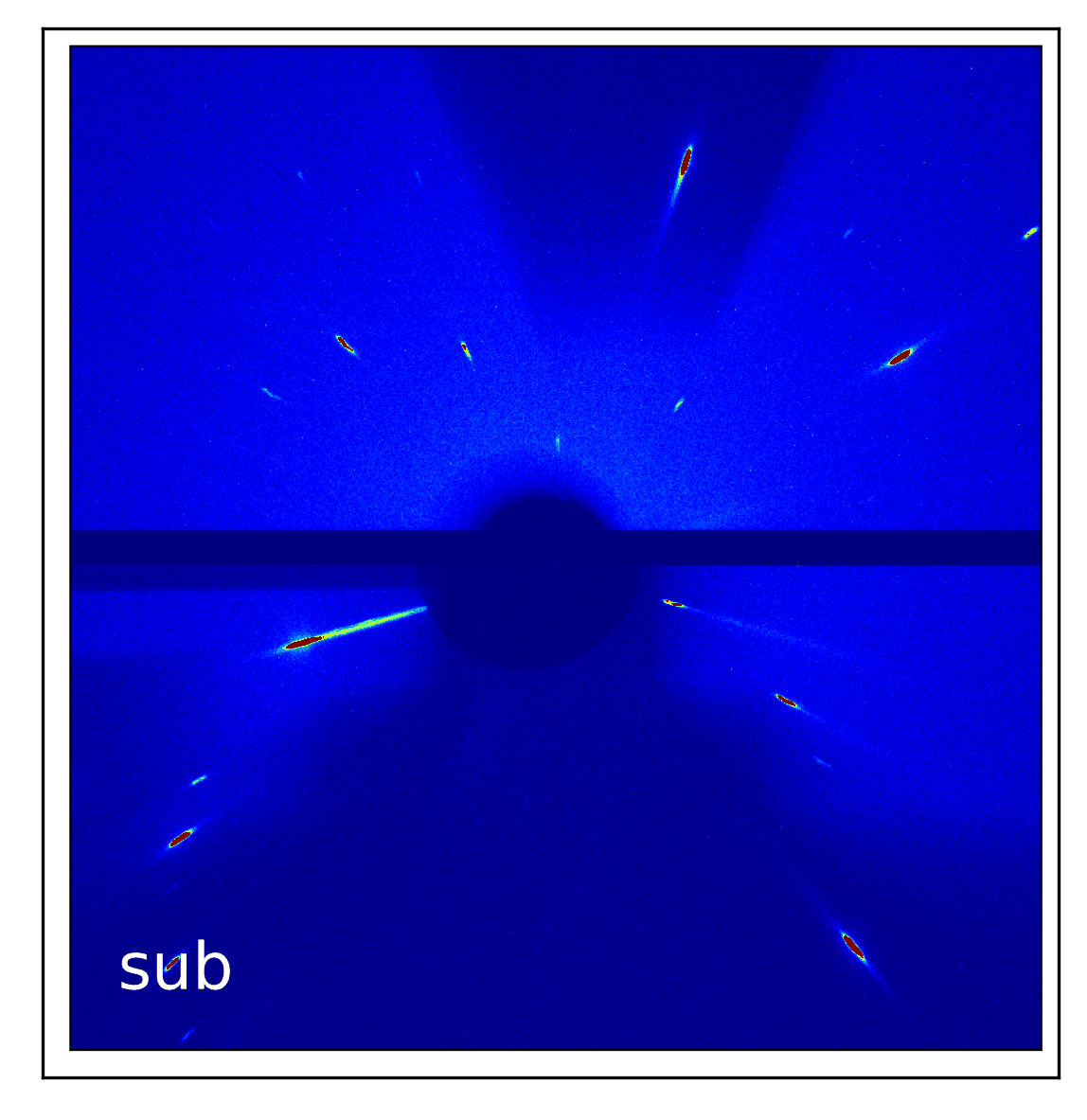

Supplement: Supplementary file 9 — Supporting file 9: advs74236‐sup‐0009‐Movies.zip. [file ADVS-13-e22704-s005.zip › Movies S3.gif]
